# De novo variants in *NPTN* cause a neurodevelopmental disorder with autism and neuroplastin-PMCA hypofunction

**DOI:** 10.1186/s13073-026-01699-7

**Published:** 2026-07-01

**Authors:** Yi Liang, Rodrigo Ormazabal-Toledo, Harini Srinivasan, Ayse Malci, Waldo Acevedo, Ulrich Thomas, Julie S. Cohen, Nils Rahner, Johannes Luppe, Gabriella Vera, Francois Lecoquierre, Eden Kroin, Brad Angle, Hong Cui, Maria J. Guillen Sacoto, Bert B. A. de Vries, Rolph Pfundt, Gillian Prinzing, Kimberly Wiltrout, Yakira Begun, Elaine M. Pereira, Alexandra Afenjar, Caroline Nava, Konrad Platzer, Dirk Montag, Rodrigo Herrera-Molina

**Affiliations:** 1 Neurogenetics Laboratory, Leibniz Institute for Neurobiology, Brenneckestrasse 6, 39118 Magdeburg, Germany; 2 State Key Laboratory of Eye Health, Eye Hospital, Wenzhou Medical University, Wenzhou, 325027 China; 3 Departamento de Química Orgánica y Fisicoquímica, Facultad de Ciencias Químicas y Farmacéuticas, Universidad de Chile, Independencia, Santiago, Chile; 4 Laboratory of Neuronal and Synaptic Signals, Leibniz Institute for Neurobiology, Magdeburg, Germany; 5 Department of Genetics and Molecular Neurobiology, Institute of Biology, Otto-Von-Guericke University, Magdeburg, Germany; 6 Center for Neuroscience Research, Children’s National Medical Center, Washington, DC USA; 7 Instituto de Química, Facultad de Ciencias, Pontificia Universidad Católica de Valparaíso, Valparaíso, Chile; 8 Center for Interdisciplinary Research in Biomedicine, Biotechnology and Well-Being (CID3B), Pontificia Universidad Católica de Valparaíso, Valparaíso, Chile; 9 Department Cellular Neurosciences, Leibniz Institute for Neurobiology, Magdeburg, Germany; 10 Department of Neurology and Developmental Medicine, Kennedy Krieger Institute, Baltimore, MD 21205 USA; 11 Department of Neurology, Johns Hopkins University School of Medicine, Baltimore, MD 21287 USA; 12 MVZ Institute for Clinical Genetics and Tumor Genetics, Bonn, Germany; 13 Institute of Human Genetics, University of Leipzig Medical Center, Philipp-Rosenthal-Str. 55, 04103 Leipzig, Germany; 14 Department of Genetics and Reference Center for Developmental Disorders, University Rouen Normandie, Inserm U1245 and CHU Rouen, 76000 Rouen, France; 15 Division of Genetics, Advocate Children’s Hospital, Park Ridge, IL 60068 USA; 16 GeneDx, LLC, Gaithersburg, MD 20877 USA; 17 Department of Human Genetics, Radboud University Medical Center, Nijmegen, the Netherlands; 18 Department of Neurology, Boston Children’s Hospital, Boston, MA USA; 19 Department of Pediatrics, Division of Clinical Genetics, Columbia University Irving Medical Center, New York, NY 10032 USA; 20 Department of Pediatrics, Division of Clinical Genetics, Columbia University Irving Medical Center and NewYork Presbyterian, New York, NY 10032 USA; 21 Genetics Department, Reference Centre for Cerebellar Malformations and Congenital Diseases and Molecular Neurogenetics Laboratory, AP-HP, Sorbonne University - Armand-Trousseau Children’s Hospital, 75012 Paris, France; 22 Sorbonne Université, Institut du Cerveau—Paris Brain Institute—ICM, Inserm, CNRS, APHP, Hôpital Pitié-Salpêtrière, Paris, France; 23 Department of Pharmacology & Physiology, ces, George Washington University, School of Medicine & Health Scien, Washington, DC USA; 24 Centro Integrativo de Biología y Química Aplicada, Universidad Bernardo O’Higgins, General Gana 1702, 8320000 Santiago, Santiago, Chile

**Keywords:** Autism, Epilepsy, Speech delay, Neuroplastin, PMCA, Calcium homeostasis

## Abstract

**Background:**

*NPTN* encodes human neuroplastin (hNp), a transmembrane immunoglobulin (Ig)-superfamily glycoprotein and a subunit of the plasma membrane calcium (Ca^2+^)-ATPases (PMCA). The critical importance of hNp and its associations with PMCA in the human brain remains unknown.

**Methods:**

Here, we describe de novo* NPTN* variants in individuals with autism and mild-to-severe DD/ID and evaluate their effects using animal models and in silico, molecular, and cellular approaches.

**Results:**

Four individuals present variants affecting the two hNp isoforms, hNp55 and hNp65. Other four variants affect only the hNp65 isoform. Two individuals independently carry the same loss-of-function nonsense variant, predicted to cause haploinsufficient production of all hNp isoforms. Haploinsufficient *Nptn*^+/–^ mice displayed reduced levels of Np and PMCA and exhibited altered social behavior. Insufficient Np55/65 production in neurons resulted in reduced PMCA expression and function. Two missense variants caused particular structural and thermodynamic abnormalities and lower expression of hNps in human embryonic kidney (HEK) cells. In primary neurons, these hNp variants failed to regulate cytosolic Ca^2^⁺ transients. In *Drosophila*, a missense mutation affecting the PMCA interaction failed to prevent the lethal phenotype caused by hNp ortholog elimination.

**Conclusions:**

We show that a novel neurodevelopmental disorder characterized by intellectual disability and autism originates from haploinsufficient *NPTN* gene dosage or insufficient functionality of mutant hNp related to PMCA hypofunction.

**Supplementary Information:**

The online version contains supplementary material available at 10.1186/s13073-026-01699-7.

## Background

Developmental delay (DD), often linked to intellectual disability (ID) and autism spectrum disorder (ASD) [1], has a prevalence of 1–3% which may vary from large international cohorts to local populations [1–3]. Due to its heterogeneity, the etiology of DD is not always understood, and it remains uncertain for 60% of the affected children [1, 3]. Exome and genome sequencing are being used to identify genetic variants as the plausible cause of DD, revealing that 40–60% of individuals with undiagnosed DD and 30–39% of the ASD cases may carry pathogenic de novo variants [3–5]. Discovering genetic variants associated with ID and ASD will facilitate early diagnosis and open avenues for their treatment.

*NPTN* encodes distinct isoforms of the type I transmembrane glycoprotein neuroplastin expressed in the brain, human neuroplastin55 (hNp55) and neuron-specific human neuroplastin65 (hNp65) [6]*.* High levels of neuroplastin mRNAs are detected at 19–24 post-coital weeks (PCW) in neurons across fetal brain regions [7], with peak levels in the prefrontal cortex of 18-year-old individuals [6]. The isoforms hNp55 and hNp65 are enriched in human synapses [6]. *NPTN* is often deleted or duplicated in individuals with 15q24 microdeletion syndrome [8, 9]. Also, a single nucleotide polymorphism in the *NPTN* promoter is associated with thinner frontal and temporal lobes in the left hemisphere of the brain, correlating with intellectual and verbal and non-verbal abilities in adolescents [10]. Studies in *Nptn*^*−/−*^ mutant mice provide direct evidence for the necessity of normal Np55/65 expression for multiple cognitive functions [6, 11].

Neuroplastin55/65 are obligatory binding partners in protein complexes for more than 95% of the four plasma membrane Ca^2^^+^-ATPases (PMCA1-4) [6, 12, 13]. The abundance and importance of these associations remain unexplored in the human brain. PMCA1-4 are ATP-fed Ca^2+^-H^+^ co-transporters that pump Ca^2+^ ultrafast toward the extracellular space to reinstate resting cytosolic levels [14, 15] and regulate intracellular Ca^2+^ signaling. Also, PMCA1-4 contribute in setting the perisynaptic alkaline pH necessary for the activation of ionotropic glutamate receptors of the N-methyl-D-aspartate (NMDA) type (iGluNRs) [16–20]. Mutations in the PMCA1-4 genes (*ATP2B1-4*) in individuals with DD, ASD, and other neurodevelopmental disorders decrease the expression and/or activity of these Ca^2+^ pumps, leading to defective restoration of cytosolic Ca^2+^ levels [21–32]. Importantly, the expression, stabilization, and activity of PMCA1-4 are strongly dependent on Np55/65 binding [6, 12–15, 33, 34] and constitutive and inducible *Nptn*-deficient mice display massive PMCA1-4 loss associated with cognitive impairments and deficits in social and affective behaviours [6, 11].

Here, we report mild-to-severe DD/ID and autism in individuals with de novo variants (missense, nonsense, or frameshift) in *NPTN*. The missense variants cause structural abnormalities in hNp isoforms as evaluated in silico through protein modelling and molecular dynamics. We confirmed that, in a human cell line, cultured primary rodent neurons, and in vivo in *Drosophila melanogaster,* the missense variants are inefficiently expressed and inefficiently support PMCA levels, resulting in failed cytosolic Ca^2+^ regulation. Haploinsuffient *Nptn*^+*/*−^ mice express reduced amounts of both Np and PMCA. In a social behavior test, *Nptn*^+*/*−^ mice display loss of preference for a novel mouse representing an endophenotype analog to social deficits that characterize autism. We conclude that the de novo variants in *NPTN* cause a neurodevelopmental disorder likely through Np-PMCA hypofunction and Ca^2+^-deregulation in central neurons.

## Methods

### Recruitment of affected individuals and animal experimentation

This investigation was approved by the ethics committee of the University of Leipzig (402/16-ek). The referring physicians obtained written informed consent for the described molecular genetic testing and permission for publication of the data from all individuals and/or their legal representatives according to the guidelines of the ethics committees and review boards of their respective institutions. Genotypic and phenotypic information was obtained from the referring physicians using a standardized questionnaire. The compilation of the individuals was supported internationally by online matchmaking via GeneMatcher [35]. Heterozygous neuroplastin-deficient mice *Nptn*^+*/*−^ were described [11]. Mice were kept with a 12 h light/dark cycle and food and water ad libitum. Animal husbandry, behavioral tests, euthanasia methods, and tissue collection were conducted in accordance with German (Tierschutzgesetz TierSchG) and European legislations (European Communities Council Directive (2010/63/EU) for the care of laboratory animals) and with the respective legal and ethical approval by the legal authorities (Landesverwaltungsamt Halle, Sachsen-Anhalt, Germany).

### Identification of *NPTN* variants

Trio exome sequencing was performed for all affected individuals and their parents, except for individuals 3 and 5 (singleton exome only). All individuals were analyzed in the context of local diagnostic protocols. As there was no causative variant identified in a known rare disease gene, and thus all individuals lacked a definite diagnosis, research evaluation of the sequencing data was conducted to potentially identify causative variants in candidate genes. The gnomAD v4.1.1 dataset served as the control population [36]. There were no significant findings other than the described variants in *NPTN*, which could explain the phenotypes of the respective individuals. All variants described were aligned to hg38, mapped to the *NPTN* MANE Select transcript NM_012428.4 and classified according to the ACMG criteria [37, 38]. *NPTN* gene sequences used for comparison are *Homo sapiens*, NP_001154835; *Macaca fascicularis*, XP_005560051; *Mus musculus*, NP_001392991; *Rattus norvegicus*, NP_001400276; *Equus caballus*, XP_023509800; *Danio rerio*, NP_991268.

### In silico prediction

In silico predictions of the missense variants were assessed using CADD-v1.6n [39], REVEL [40], MutPred2 [41], VEST4 [42], and BayesDel [43] using cutoffs for deleterious predictions from Pejaver et al. [44].

### Molecular dynamics and docking

Molecular dynamics simulations were performed with Gromacs 2020.3 [45] and the OPLS-AA force field [46]. The models were solvated in a cubic box with the SPCE water model, and NaCl counterions were added to neutralize the system [47]. System energy was minimized with the steepest descent algorithm up to a convergence criterion of 1000 kJ mol^–1^nm^–1^. To equilibrate temperature and pressure, we performed two consecutive equilibration steps of 200 ps, first in the NVT ensemble and then in the NPT ensemble. After that, a 100ns production run in the NPT ensemble was performed to obtain geometrical information on all variants considered. Hydrogen bonds restraining was performed during equilibration and production runs using the LINCS algorithm [48]. Long-range electrostatic interactions were treated using the PME method (cutoff of 10Å) [49]. Temperature was kept constant (300 K) using the V-rescale thermostats and pressure (1 bar) using the Parrinello-Rahman barostat [50, 51]. The atomic coordinates of hNp65^wt^ were taken from our previous work [52]. The P342L variant was generated from hNp65^wt^ structure using the Rosetta fixbb protocol [53] with the default REF2015 score function [54] allowing side-chain replacement and rotamer repacking on a fixed protein backbone. For structural visualization, the lowest-scoring mutant model was used [55]. For the analysis of hNp65^p.W135R^, we used the last frame obtained from the molecular dynamics simulation to compare Np65 dimers as described elsewhere [56]. Crystallographic information described elsewhere was used for the docking of hNp65^p.P342L^ to hPMCA1 [33] or hPMCA2 [14], which was performed directly from the structure obtained with Pymol, because this amino acid change occurs within the intermembrane space. The binding interfaces with low-energy conformations were identified using the HADDOCK docking protein–protein web server [57, 58].

### Cell cultures

Human embryonic kidney cells (HEK293T) were prepared in Dulbecco’s Modified Eagle Medium (DMEM, Gibco) with 1% penicillin/streptomycin (Gibco), 1% L-glutamine (Gibco) and 10% fetal bovine serum (Gibco) at 37 °C, 5% CO_2_ [6]. Primary hippocampal neurons were prepared from E16-18 rat embryos following our published protocols [6, 34, 52, 59]. In short, neurons were dissociated with trypsin-EGTA for 15 min at 37°C. 50,000 neurons were plated on poly-D-lysine-coated glass coverslips for 12-well plates. After one hour, when the cells were well-attached to the coverslips, the plating medium DMEM 1% penicillin/streptomycin, 1% L-glutamine, and 10% horse serum was replaced with 1 ml of Neurobasal medium with 2% B27 supplement (Thermo Fisher Scientific Inc.), 1% penicillin/streptomycin, and 1% L-glutamine. At day 7 in vitro, 100 μl of fresh culture medium was added. After growing and developing, cultured neurons were transfected at days 10–11 in vitro and used at days 14–16 in vitro (see below).

### Constructs, plasmids, siRNA, and transfections

Constructs of human Np wild-type (hNp65: NM_012428.4, hNp55: NM_017455.4; Additional File 1: Fig. S1) and human PMCA2 (hPMCA2; Addgene, ID: 47584, Cambridge, MA) have been characterized [6]. TagRFPT-GCaMP5G plasmid has been described [34]. PGP-CMV-jGCaMP7f plasmid was obtained from Addgene (ID: 104483). We used control (scrambled) siRNA duplexes and siRNA targeting all premature Np RNA (Santa Cruz Biotechnology, Inc., ID: sc-149938). *NPTN* missense variants were generated by PCR amplification (Additional File 1: Table A). DNA-fragments were inserted into linearized vector FUGW (Addgene, ID: 14883) using cold-fusion cloning (System Biosciences, Palo Alto, CA). Transfections were performed with Lipofectamine 2000 following the company’s protocol (Invitrogen, Darmstadt, Germany). Hippocampal neurons were transfected with plasmids (1 µg/well) with or without control (scrambled) siRNA or Np siRNA (0.1–1 µg) at days 10–11 in vitro. 300,000 HEK293T cells were transfected with plasmids (1.5 µg/well) 24 h after seeding in 6-well plates and harvested 24 h later.

### Western blot

HEK293T cells were harvested with lysis buffer (1% Triton X-100 in 50 mM Tris/HCl, pH 8.0) supplemented with protease inhibitor cocktail (cOmplete™ ULTRA tablets, Roche), homogenized using an ultrasonic homogenizer, and spun down at 12.000g for 20 min. The supernatant was collected and mixed uniformly with 2X SDS loading buffer and boiled at 95 °C for 5 min. Proteins were separated by sodium dodecyl sulfate–polyacrylamide gel electrophoresis (SDS-PAGE) on 10% gels and electro-transferred to a nitrocellulose membrane (Cytiva, AmershamTM ProtranTM 0.45µm NC). After blocking with 5% nonfat milk in TBS-T solution, TBS containing 0.1% Tween 20 for 1 h, the membrane was incubated with primary antibodies (Additional File 1: Table B) overnight at 4°C. Afterwards, the membrane was washed three times with TBS-T and incubated with secondary antibodies for 1 h at room temperature. After washing, the membrane was incubated with Immobilon Western Chemiluminescent HRP Substrate (EMD Millipore) and chemiluminescence was detected using Intas Chemocam ECL Imaging system.

### Immunocytochemistry

Cultured neurons were fixed and stained according to established protocols [6, 34, 52]. Briefly, cultures were fixed with 4% paraformaldehyde (PFA) containing 2% sucrose in 1X PBS for 8–10 min, washed carefully with 1X PBS and incubated with blocking solution (10% horse serum and 0.1% Triton X-100 in PBS) two times, 20 min each. Then they were incubated for one hour with primary antibodies (Additional File 1: Table B) mixed with the blocking solution. After incubation, the coverslips were washed 3 times with 1X PBS for 10 min each. Secondary antibodies (Additional File 1: Table B) diluted in the blocking solution were added to the coverslips and incubated for 1 h. The coverslips were then washed 3 times with 1X PBS and mounted on microscopy slides with Mowiol.

### Confocal microscopy and image quantification

Images of neurons were acquired using an oil-immersion objective HCXAPO 63X/1.40 NA coupled to an upright confocal microscope TCS SP5 (Leica), with a pinhole value of 0.75 AU, under sequential scanning mode (200 Hz), with 1-, 3- or sixfold digital magnification for the whole cell, cell soma, or dendrites, respectively, and digitized in a 1054 X 512 format.

### Calcium imaging

Ca^2+^ imaging was performed in transiently transfected primary hippocampal neurons at 14–16 days in vitro following our published procedures [34, 52]. Glass coverslips were carefully placed into an imaging magnetic chamber equipped with two silver wires for field electrical stimulation (Warner Instruments, Hamden, CT) and then filled with 1 ml of Tyrode’s solution. Stimulation was 10 or 20 biphasic pulses (1 ms duration each) generated with a S48 stimulator (Astro-Med, Inc., West Warwick, RI, USA). Evoked Ca^2+^ transients were recorded using an inverted microscope Observer D1 (Zeiss, Jena, Germany) with a 63X/1.20 NA objective and an EMCCD camera Evolve 512 (Delar Photometrics, Tucson, AZ) under the control of VisiView software (Visitron Systems GmbH, Puchheim, Germany). Fluorescence intensity changes were quantified using Fiji/ImageJ software and parameters were extracted using pCLAMP 10 (Molecular Devices, San Francisco, CA).

### *Drosophila melanogaster* studies

UAS-hNp55 transgenic constructs were established in the vector pJFRC12 (Addgene clone 26222) and used for PhiC31-mediated germline transformation into the attP40 target site on the second chromosome (cytological position 25C6) of the recipient strain (y1 w67c23; P[CarryP]attP40). This procedure was performed by BestGene (Chino Hills, CA). Transgenic flies were identified based on orange eye color and established as stocks carrying the CyO^GFP^ balancer chromosome. For further details on the transgenic lines see Additional File 1: Generation and imaging of mutant *Drosophila melanogaster*.

### Social interactions of Nptn-deficient mice

Social interactions of *Nptn*^+/–^ and *Nptn*^+*/*+^ adult male matched littermate mice were analyzed using the three-chamber test as described [60]. Briefly, during 3 test phases (10 min each), the mouse could explore all compartments. In phase 1, the mouse was alone for habituation. In phase 2, an unfamiliar C57BL/6NCrl wild-type mouse (male, novel 1) was placed in one of the wire cups. In phase 3, another unfamiliar C57BL/6NCrl mouse (same sex, novel 2) was added to the other cup. Time spent in each compartment, in contact with strangers, and transitions between compartments were recorded.

### Statistical analysis

For Ca^2+^ imaging, a Mann–Whitney U test was used for group comparisons for non-parametric data with sample sizes > 20, and a Wilcoxon matched-pairs test for the inner group comparison. Statistical analysis of Ca^2+^ imaging data was performed using Prism9 software (GraphPad), with outliers from raw data screened out with the Grubbs’ test (Q = 1). For Western blots and Immunocytochemistry, statistical analysis of optical density measurement data used Student’s *t*-test. For the analysis of behavior, Statview (SAS Institute Inc., Cary, NC) was used for analysis of variance (repeated measures ANOVA) and post hoc analysis (Scheffe’s test). *P*-value smaller than 0.05 (*P* < 0.05) was considered statistically significant.

## Results

### Clinical and genetic analysis

**Fig. 1:**
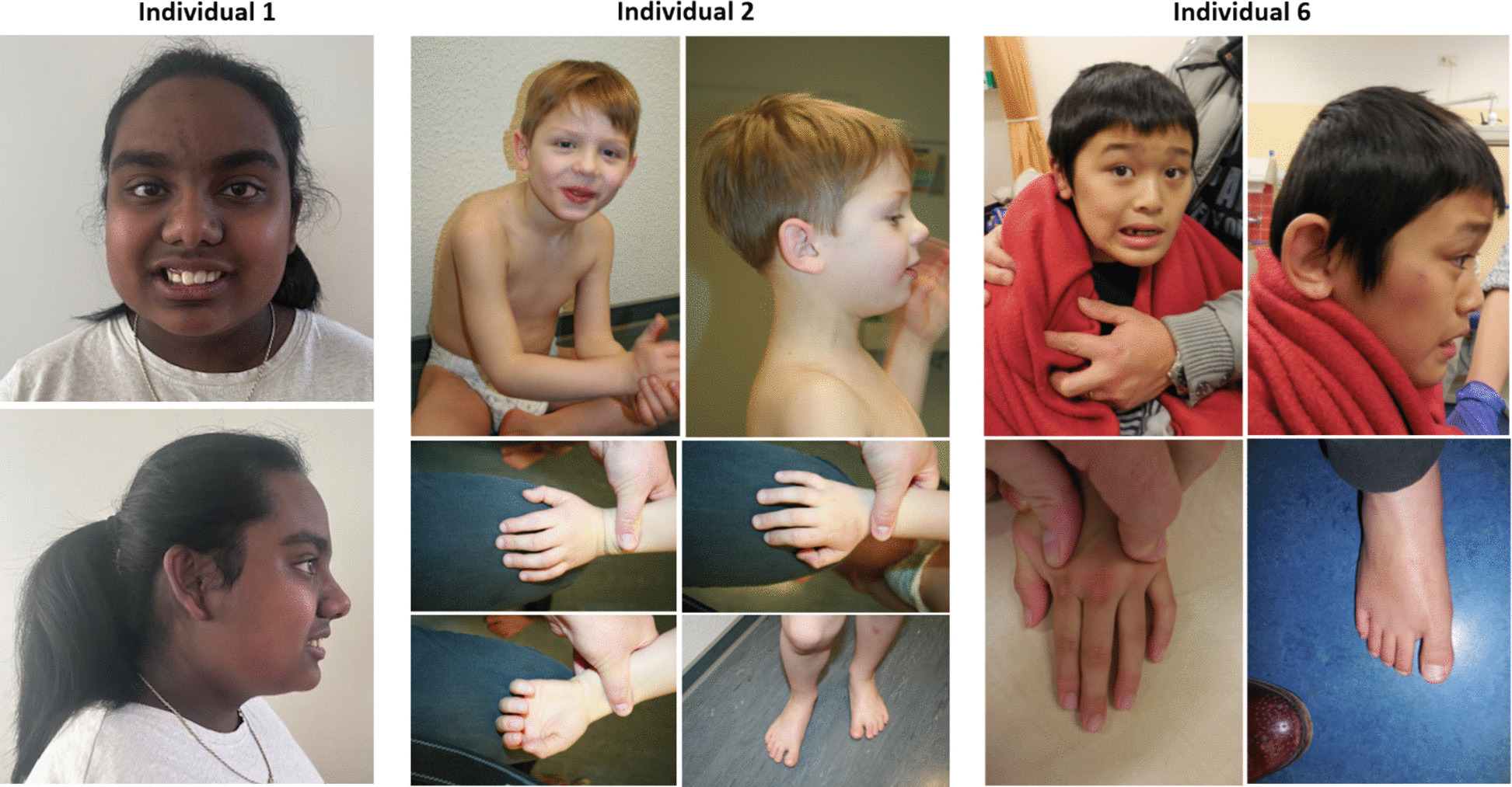
*NPTN* individuals. Photographs of three individuals carrying missense (individuals 1 and 2) or frameshift (individual 6) de novo variants. These individuals are characterized along other individuals in Table 1 and described in the main text

We describe a cohort of eight individuals with heterozygous variants in *NPTN*, seven of which are of de novo origin. An overview of the clinical evaluation of all individuals is presented in Table 1. Additional descriptions are provided as case reports in the Additional file 1: case reports and in Additional file 2: Table S3 and Table S4. All eight individuals presented with developmental delay (DD) and/or intellectual disability (ID) ranging from mild to severe. Four individuals displayed severe, two moderate, and two mild DD/ID, respectively. Seven individuals were diagnosed with autism spectrum disorder. Other behavioral findings included poor social interactions, a high pain threshold, automutilation or repetitive behaviors. Individual 1 presented with seizures starting at age seven months with epileptic spasms. That same individual developed focal impaired awareness seizures at eight years of age and was not seizure-free at age 17. Five of the seven individuals received a cranial MRI. Of note, individual 3 presented with vermal dysgenesis. Individual 4 was reported to have a period of significant regression of speech skills. Growth was found normal in all individuals. Subtle dysmorphic facial features were reported in six out of eight individuals and upslanting palpebral fissures and a prominent forehead were recurrently observed in individuals 1, 2, and 6 (Fig. 1).

**Table 1:** Clinical and genetic details of all affected individuals with causative variants in *NPTN*

**Ind**	**Age (Sex)**	**Variant (NM_012428.4)**	**DD/ID**	**autism, behaviour, neurological findings**	**Dysmorphic features**	**Further findings**
1	17y (F)	c.403 T > A, p.(Trp135Arg), de novo	moderate	autism, epileptic spasms (onset 7 months); focal impaired awareness seizures (onset 8 years); still occasional seizures	upslanting palpebral fissures, slight ectropion, prominent forehead with frontal upsweep and high anterior hairline	no
2	7y (M)	c.1025C > T, p.(Pro342Leu), de novo	severe	autism, repetitive behaviour, tics (DD dystonia), no speech	mild hypertelorism, downslanting palpebral fissures, full lips, Darwin tuberculum on both ears, fetal pads, sandal gaps	recurrent diarrhea, macrocephaly
3	5y (F)	c.14C > A, p.(Ser5*), de novo	severe	autism, poor social interactions, repetitive behaviour, hypotonia, ataxic gait	downslanting corners of the mouth, anteverted nares, bilateral epicanthus	joint hyperlaxity, chronic constipation, bilateral vesicoureteral reflux
4	6y (M)	c.218_219del, p.(Glu73Valfs*53), de novo, mosaic	severe	autism, period of regression	no	allergies, eczema, hyperropic astigmatism
5	3y (F)	c.284C > G, p.(Ser95*), heterozygous	mild	autism, poor social interactions, ritualistic behaviour, mild cerebral palsy in lower extremities	no	laryngomalacia
6	2y (M)	c.342C > G, p.(Tyr114*), de novo	moderate	autism, poor social interactions, hyperactivity, possible speech regression	prominent forehead	early eruption of teeth, insomnia
7	9y (M)	c.902del, p.(Asn301Thrfs*3), de novo	severe	tentative diagnosis of autism, pica disorder, automutilation, high pain threshold	flat face, full eyebrows, upslanting palpebral fissures, bilateral epicanthus, long fingers with mild clinodactyly 5th fingers, normal feet with sandal gaps	no
8	7y (F)	c.14C > A, p.(Ser5*), de novo	mild	hyperphagia	hypertelorism, bilateral epicanthus, short fingers	sleep apnea

Further clinical details are provided in Supplementary data and Supplementary Table 1

*Abbreviations*: *DD* developmental delay, *F* female, *Ind*. Individual, *ID* intellectual disability, *M* male, *y* years

### Genetic results

Trio exome or genome sequencing revealed de novo variants in *NPTN* in individuals 1, 2, 4, 6–8. A single exome test was carried out in individuals 3 and 5. The nonsense variant in *NPTN* in individual 3 was segregated in the parents with Sanger sequencing and confirmed a de novo origin. The biological parents of individual 5 were not available for testing. Variants in individuals 3 and 8 are identical. All variants are not recurrent and are absent from gnomAD (v4.1.1 dataset). Two distinct de novo missense variants were identified in addition to five predicted loss-of-function (pLoF) variants. Multiple in silico tools predict a deleterious effect of the two *NPTN* missense variants (Additional file 2: Table S3 and Table S4). Missense variants as well as pLoF variants are highly depleted from the gnomAD database. This indicates a selective constraint on both types of variants in a general population that lacks severe, early-onset phenotypes such as DD and ID (LOEUF = 0.25; pLI = 0.1; o/e for missense variants = 0.58; z-score = 4.06).

### Characterization of the *NPTN* variants

**Fig. 2:**
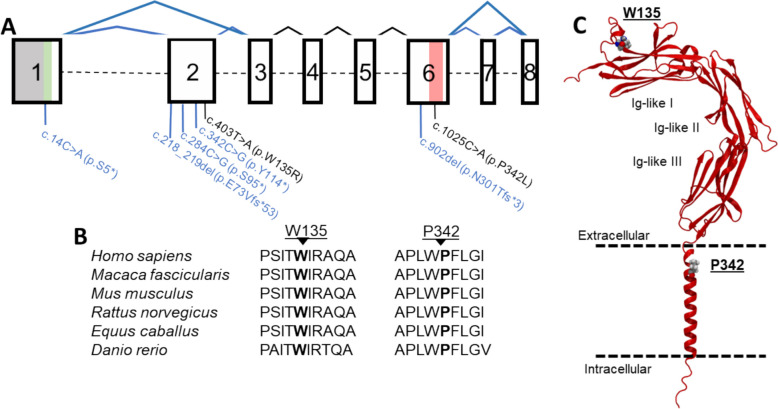
*NPTN* monoallelic variants. **A** Diagram of the *NPTN* gene and localization of missense (black font) and frameshift and nonsense (blue font) variants identified in the individuals. The *NPTN* gene contains eight exons with obligatory (black lines) and alternative (blue lines) splicing points and yields two hNp isoforms (hNp55 or hNp65). In exon 1, the gray band represents the 5’ untranslated region and the light green band the signal peptide sequence. The splicing of exon 1 to exon 3 results in the elimination of exon2 encoding the hNp65-specific Ig-like domain I and leads to the synthesis of hNp55. Exons 3–5 encode Ig-like domains II and III and exon 6 encodes a single transmembrane domain (light red band) common to all hNp isoforms. The small exon 7 can be removed from hNp55 and hNp65 mRNAs producing proteins with cytoplasmic tails lacking a four amino acidic DDEP insert. **B** Missense variants substitute conserved amino acids located in highly conserved amino acidic sequences across different species. **C** The localization of the missense variants is displayed in the protein structure of hNp65. Ig: immunoglobulin

We located in the genomic sequence of *NPTN* the position of each of the variants identified (Fig. 2A). *NPTN* consists of eight coding exons and one non-encoding 3'UTR exon. Alternative splicing of exon 2, encoding Ig-like domain I (Ig-like I), results in mRNAs for the translation of either of the two main glycoprotein isoforms hNp55 (containing Ig-like II and III) or hNp65 (containing Ig-like I, II, and III). Np55 and Np65 may display an alternative aminoacidic motif (DDEP) encoded by the mini-exon 7 [61, 62]. The function of the DDEP insert is unknown; however, it has been reported that its presence or absence does not affect the expression of the Np isoforms or Nps’ functions in the regulation of PMCA levels, synaptogenesis, or cell signalling [13, 59]. Therefore, hNp65 is 394 or 398 amino acids long with three extracellular Ig-like domains encoded by exons 1–6, a single transmembrane domain (TM) encoded by exon 6, and a 34 or 38 amino acids long intracellular domain encoded by exons 6–8. Np55 is 278 or 282 amino acids long and distinctly displays only the Ig-like domains II-III.

The two *NPTN* missense variants reported here affected conserved amino acids located within conserved amino acid sequences in different vertebrate species (Fig. 2B), *C. elegance* (Additional file 1: Table S2), and *D. melanogaster* (Fig. 4). The missense variant c.403 T > A (p.W135R), the nonsense variants c.284C > G (p.S95*) and c.342C > G (p.Y114*), and the frameshift variant c.218_219del (p.E73Vfs*53) are located at the hNp65-specific exon 2 (Fig. 2A and C). Other variants affect exons 1 or 6 which are common to both hNp isoforms (Fig. 2A and C). Interestingly, the missense variant c.1025C > A (hNp65: p.P342L; hNp55: p.P226L) replaces a key amino acid in the transmembrane domain of hNp55/65 that interacts directly with PMCA [14, 15]. The nonsense variant c.14C > A (p.S5*) stops the translation of the signal peptide, thus the synthesis of Np55 and Np65. The nonsense variants p.S95* and p. T114* stop hNp65-specific mRNA translation, resulting in truncated Ig-like I domains with antiparallel *β*-sheet structures and without functional modules (Additional file 1: Fig. S2). Also, these two truncated Ig-like I domains will lack the obligatory cysteine52–cysteine116 disulfide bond that maintains the folded Ig-like structure [56]. Whereas the frameshift variant p.E73Vfs*53 is located at the beginning of the hNp65-specific exon 2, the frameshift variant c.902del (p.N301Tfs*3) is located in the exon 6 common for hNp55 and hNp65. These two variants shift the reading frame to produce a premature stop-codon in the affected mRNA and thus, they may result in truncated extracellular domains with unknown stability. In silico analysis of mRNA stability, validated in previous studies [63, 64], indicates that the mRNAs of p.E73Vfs*53 and p.N301Tfs*3 are subject to degradation by nonsense-mediated decay (Additional file 1: Fig. S1) and thus anticipates a potentially very low number of these mRNAs available for encoding for the putative truncated extracellular domains.

We observed that *NPTN* variants carried by the individuals 2, 3, and 7 with severe DD/ID affect both hNp55 and hNp65 and that the individuals 1, 5, and 6 with moderate/mild DD/ID compromise only Np65. The individual 4 appears to escape from this categorization. The individual 8, carrying the same variant as individual 3, shows mild DD/ID. These observations suggest a potential correlation between the phenotype severity and haploinsufficiency of only Np65 *vs.* both Np55 and Np65, which is observed for the phenotypes of *Nptn*65-specific [65] *vs. Nptn*^*−/−*^ mice [11].

### Computational analysis of the *NPTN* missense variants

**Fig. 3:**
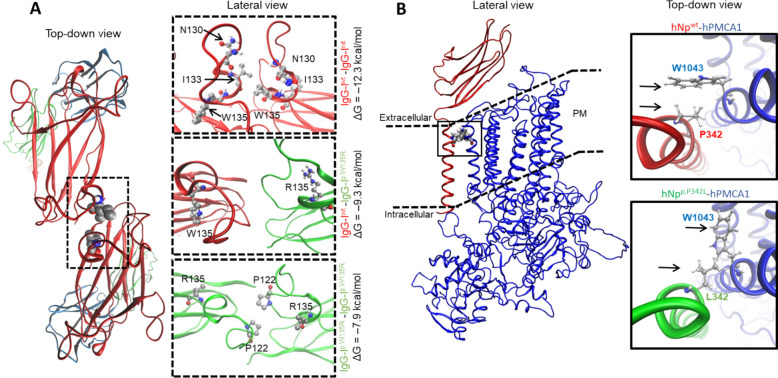
Structure of the proteins resulting from *NPTN* missense variants from individual 1 (A, p.W135R) and individual 2 (B, p.P342L). **A** Views and ΔG binding of each of the *trans*-homophilic interactions were extracted from our molecular docking simulations and based on previous kinetic and crystallography studies. hNp65^wt^ (red) and hNp65^p.W135R^ variant (green). The sequential replacement of hNp65^wt^ by hNp65^p.W135R^ results in severe conformational changes with energetically less favorable and more unstable dimerization. Arrows indicate distances between the key amino acids. **B** Interactions resulting from molecular docking simulations between the transmembrane domain 10 (T10) of hPMCA1 (blue) with the transmembrane domain of hNp^wt^ (red) or with hNp^p.P342L^ (green) are based on the refined structural interpretation informed by the higher-resolution PMCA2–Nptn structure of Vinayagam et al. [14] and crystallography of the hNp-hPMCA1 complex [33]. PM: plasma membrane. Arrows indicate that the distance necessary for the interacting amino acids P342 in hNp^wt^ and W1043 in hPMCA1 is drastically reduced for the pair L342 in hNp.^p.P342L^ and W1043 in hPMCA1

We performed a customized structural and thermodynamic analysis of the two *NPTN* missense variants W135R and P342L using molecular dynamics and protein–protein docking modeling and calculating binding energies (*∆G* binding) (Fig. 3 and Additional file 1: Fig. S2). Based on the resolved structure of the Np65-specific Ig-like I [56], our computational procedures were robust enough to recreate the hNp65^wt^-hNp65^wt^ *trans-*homophilic binding [56] (Fig. 3A, top-down view) and showed that W135, N130, and I133 have an important participation in the thermodynamically spontaneous attraction between hNp65^wt^ Ig-like I F-G loops (Fig. 3A, upper frame in lateral view). In the variant hNp65^p.W135R^ resulting from c.403T > A, the F-G loop structure is altered and the N130 and I133 are far off from reaching effective *trans-*interaction positions (Fig. 3A, middle frame in lateral view). An increased *∆G* confirms the reduced binding efficiency of the hNp65^p.W135R^ F-G loop to form the pair hNp65^wt^-hNp65^p.W135R^. The interaction of the pair hNp65^p.W135R^-hNp65^p.W135R^ was worsened by the appearance of an abnormal P122-P122 interaction with an even higher *∆G* for their binding (Fig. 3A, middle frame in lateral view).

In agreement with the reported crystallographic structure of the hNp-hPMCA1 complex [33], we localized P342 at the hNp transmembrane domain (TMD) facing W1043 at the hPMCA TM10 and found that it participates in the stable interaction of the proteins within the cell plasma membrane (lateral view in Fig. 3B). When the hNp65^p.P342L^ variant resulting from 1025 C > A was projected onto the hNp-hPMCA1 interaction surface, we observed that the replacement of the cyclic P-ring by the aliphatic side chain in the mutant residue L342 violates the effective interaction distance with hPMCA1 TM10 W1043, creating a thermodynamic constraint that would interfere with the intermolecular interaction (lower frame in top-down view in Fig. 3B). Using the recently reported high-resolution hNp-hPMCA2 cryo-EM structures by Vinayagam et al. [14], we confirmed that the mutant interaction surface is conserved also in PMCA2 (Additional file 1: Fig. S2). Interestingly, Vinayagam et al. [14] demonstrated the existence of additional interaction contacts that may not be affected in the P342L variant (see below).

### Expression of *NPTN* missense variants and effects on PMCA levels

Based on our previous studies [6, 34, 52], we tested the expression levels of hNp65^wt^, hNp55^wt^, hNp65^p.W135R^, hNp65^p.P342L^, and hNp55^p.P226L^ in transfected HEK293T cells (Additional file 1: Fig. S3). As expected [6, 34, 52], hNp65^wt^ and hNp55^wt^ were efficiently detected by Western blot analysis (Additional file 1: Fig. S3). Importantly, decreased expression was found for hNp65^p.W135R^ (*p* < 0.001 *vs* hNp65^wt^) and hNp65^p.P342L^ (*p* = 0.033 *vs* hNp65^wt^) whereas levels of hNp55^p.P226L^ were similar to hNp55^wt^ (Additional file 1: Fig. S3). Neuroplastin is an obligatory binding partner and post-transcriptionally promotes the expression of PMCA [6, 12–15]. Therefore, we examined the effect of the missense variants on the capacity of hNp to increase hPMCA2 levels. hPMCA2 levels in hNp65^WT^- and hNp55^WT^-expressing cells were much higher than the non-transfected control cells (Additional file 1: Fig. S3). Compared to double transfected cells with hPMCA2/hNp65^WT^ or hPMCA2/hNp55^WT^, all missense variants promoted hPMCA2 inefficiently. Indeed, hPMCA2 was less in cells expressing hNp65^p.P342L^ (*p* = 0.004 *vs* hNp65^wt^), hNp65^p.W135R^ (*p* = 0.011 *vs* hNp65^wt^) or Np55^p.P226L^ (*p* = 0.057 *vs* hNp55^WT^) (Additional file 1: Fig. S3). Compared to corresponding wt controls, the ability to increase hPMCA2 (hPMCA2/hNp ratio) was reduced for hNp65^p.P342L^ and hNp55^p.P226L^ but not for hNp65^p.W135R^ (Additional file 1: Fig. S3) pointing to a specific necessity of this mutated proline residue in both hNp isoforms for normal levels of hPMCA2 expression in human cells. The residual capacity of hNp65^p.P342L^ and hNp55^p.P226L^ to promote a low expression of hPMCA2 may be related to further interaction contacts [14, 56].

**Fig. 4:**
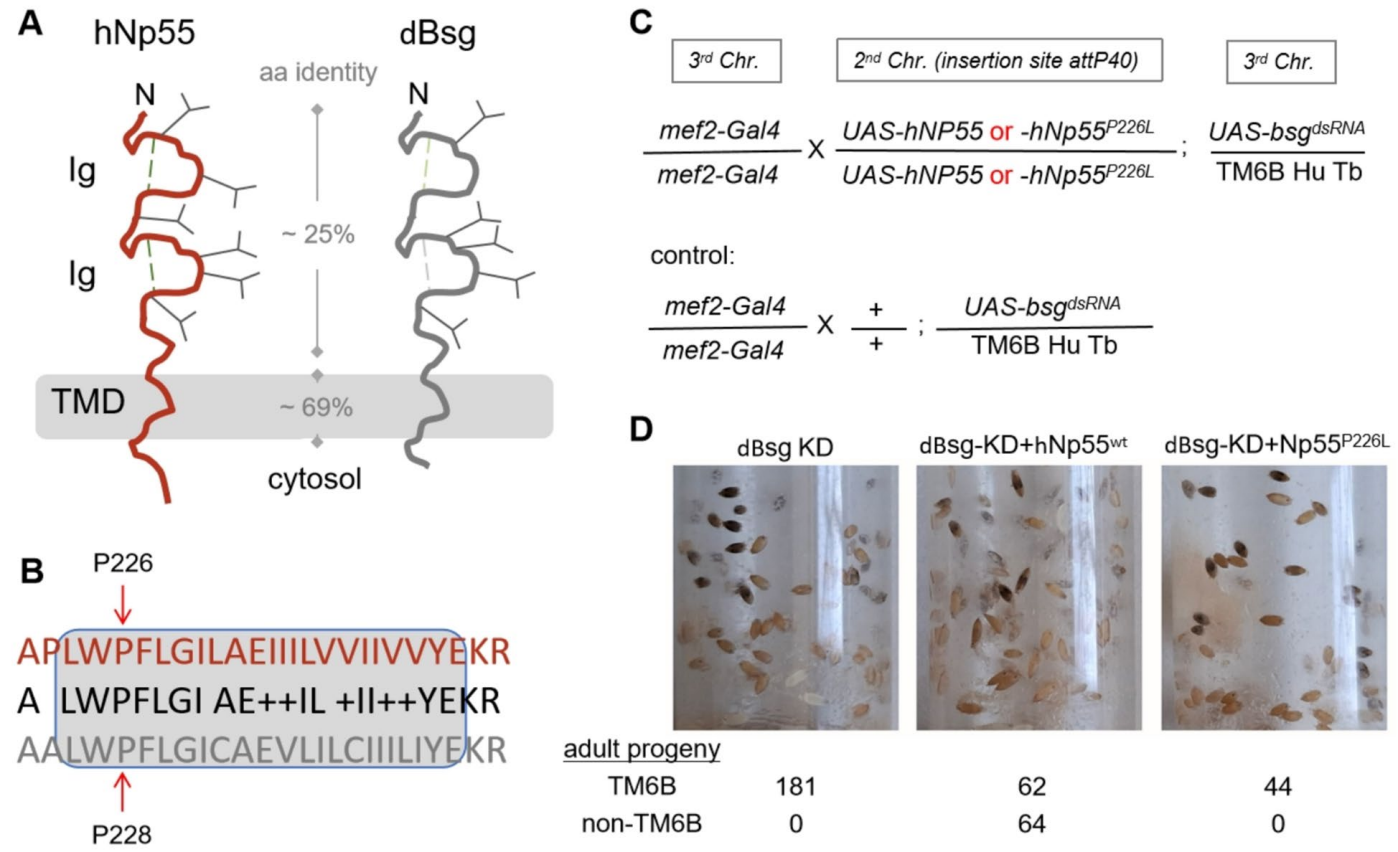
Lethality due to dBsg-KD during early muscle development is rescued by hNp55 but not hNp55^P226L^. **A**, **B** Structural homology between hNp55 (red) and dBsg (gray). **C** Crossing scheme for the assessment of the rescue capacity of hNp55 and hNpP226L during muscle development. Flies homozygous for the early-onset muscle Gal4 driver mef-Gal4 were crossed to effector lines homozygous for either UAS-hNp55 or UAS-hNp55P226L, and heterozygous for UAS-bsgdsRNA over the balancer chromosome TM6B carrying the dominant markers Humeral (Hu, visible on adults) and Tubby (Tb, visible on L3 larvae and pupae). Control crosses lacked the UAS-hNp55 or UAS-hNpP226L effectors. **D** Pupal progeny from the crosses in (C) on the wall of a culture vial. Note that in crosses with dbsg-KD alone or with dbsg-KD plus hNp55^P226L^ only round-shaped Tubby pupae are detectable, whereas crosses with dbsg-KD plus hNp55 give rise to both Tubby and normally shaped pupae. This clear discrepancy is confirmed by counts of adult progeny with TM6B or without TM6B

The impact of P226L on hNp55 functionality was further evaluated in *Drosophila melanogaster,* a classical system to study neurodevelopment and synaptic mechanism [66, 67] (Fig. 4). In contrast to the three mammalian paralogs *NPTN, BSG*, and *EMB*, only a single orthologous gene encoding dBsg exists in *Drosophila.* dBsg shows an overall 25% amino acid sequence identity and a transmembrane domain homology of 69% (including adjacent intra- and extracellular amino acid residues) with hNp55 (Fig. 4A, B). Deletion of dBsg expression in muscle is known to be lethal at the late embryonic to early larval (L1) stage [68]. Thus, we tested whether the co-expression of hNp55^wt^ or hNp55^p.P226L^ rescues the lethal phenotype triggered by dBsg knockdown due to mef2-Gal4-induced expression of dsRNA (Fig. 4C, D). As expected, dBsg knockdown caused a highly penetrant lethality around the L1 stage. The larval lethality was fully rescued by hNp55^wt^ as virtually all progeny developed into viable adult flies, indicating tolerance to the differences between dBsg and hNp55^wt^. Strikingly, hNp55^p.P226L^ displayed only minimal if any rescue capacity, as all progeny died before the L2 stage (Fig. 4D).

### Effect of *NPTN* missense variants on cytosolic Ca^2+^ regulation

**Fig. 5:**
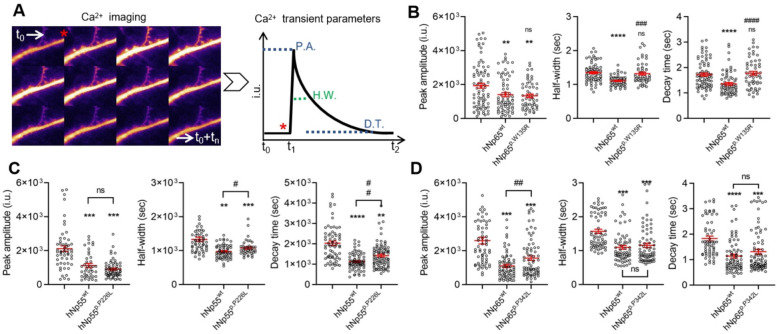
Effect of *NPTN* missense variants from individual 1 and 2 on cytosolic Ca^2+^ transients. **A** Summarized Ca^2+^ imaging sequence (1pic/400 ms) of a representative GCaMP5G-expressing secondary dendrite (100 m section) depolarized with a field electrical stimulation of 10 pulses at 20 Hz (red asterisk at t_1_). From these image sequences, the parameters peak amplitude (P.A.), half-width (H.W.), and 90% decay time (D.T.) were calculated to describe the evoked cytosolic Ca^2+^ transients [31]. **B-D** Parameters were obtained from GCaMP5G-expressing dendrites (open circles) transfected or not with one of the wt isoforms or one of the *NPTN* variants. Data from at least three biological replicates (independent cultures/preparation). For each replicate, three-four neurons were recorded. Three-four dendritic segments per neuron were analyzed as previously described [34, 43]. For data presentation clarity mean ± S.E.M. are displayed, but additionally S.D. is given for each condition. **B** For P.A.: control (no label) *n* = 81; mean = 1931 ± S.D. = 1333; hNp65^wt^ *n* = 71; 1408 ± 1006; hNp65^p.W135R^ n = 57; 1335 ± 798. **p < 0.01 unpaired *t*-test *vs.* control; ns: no significant difference; unpaired *t*-test *vs.* hNp65^wt^. H.W.: control 1349 ± 265; hNp65^wt^ 1108 ± 139; hNp65^p.W135R^ 1320 ± 306. ****p < 0.0001 or ns *vs.* control. ###*p* < 0.001 *vs.* hNp65^wt^. D.T.: control mean = 1724 ± 520; hNp65^wt^ 1359 ± 448; hNp65^p.W135R^ 1775 ± 570. ****p < 0.0001 or ns *vs.* control. ####*p* < 0.0001 *vs.* hNp65^wt^. **C** For P.A.: control (no label) *n* = 64, mean = 2116 ± 1237; hNp55^wt^ *n* = 60, 1121 ± 754; hNp55^p.P226L^ *n* = 82, 909 ± 510. ***p < 0.001 unpaired *t*-test *vs.* control. ##*p* < 0.01 or ns unpaired *t*-test *vs.* hNp55^wt^. &&*p* < 0.01 one-way ANOVA. H.W.: control 1332 ± 467; hNp55^wt^ 976 ± 199; hNp55^p.P226L^ 1079 ± 231. **p < 0.01 or ***p < 0.001 *vs.* control. #*p* < 0.05 *vs.* hNp55^wt^. D.T.: control 2034 ± 866; hNp55^wt^ 1123 ± 403; hNp55^p.P226L^ 1422 ± 600. **p < 0.01 or ****p < 0.0001 *vs.* control. ##*p* < 0.01 *vs.* hNp55^wt^. &&*p* < 0.01 one-way ANOVA. **D** For P.A.: control (no label) *n* = 64, 2597 ± 1598; hNp65^wt^ *n* = 67, 1102 ± 772; hNp65^p.P342L^ *n* = 72, 1564 ± 1133. ***p < 0.001 unpaired *t*-test *vs.* control. ##*p* < 0.01 unpaired *t*-test *vs.* hN65^wt^. &&*p* < 0.01 one-way ANOVA. H.W.: control 1574 ± 496; hNp65^wt^ 1100 ± 462; hNp65^p.P342L^ 1158 ± 552. ***p < 0.001 *vs.* control. D.T.: control 1831 ± 728; hNp65^wt^ 1137 ± 643; hNp65^p.P342L^ 1312 ± 830. ***p < 0.001 or ****p < 0.0001 *vs.* control

Neuroplastin-PMCA complexes are crucial for cytosolic Ca^2+^ extrusion and shaping of Ca^2+^ signaling in brain neurons [6, 12–15, 33, 34]. To evaluate the functional effect of *NPTN* missense variants on Ca^2+^ regulation, we investigate electrically-evoked cytosolic Ca^2+^ transients using Ca^2+^ imaging in GCaMP5G-expressing primary hippocampal neurons at days 14–16 in vitro (referred to as GCaMP5G-neurons) (Fig. 5A). We quantified peak amplitude, half-width, and decay time of the evoked Ca^2+^ transients, as these parameters reflect how Ca^2+^ transients are shaped by the levels and activity of Np-PMCA complexes in synapses and dendrites [34, 52] (Fig. 5A). In line with the previous reports showing that Np^wt^ over-expression adds on endogenous Np to promote PMCA levels and function (gain-of-function) [34, 52], GCaMP5G-neurons co-expressing hNp65^wt^ or hNp55^wt^ displayed evoked Ca^2+^ transients with smaller peak amplitudes and faster restoration of basal Ca^2+^ levels compared to control GCaMP5G-neurons (Fig. 5B-D). In contrast, hNp65^p.W135R^, hNp65^p.P342L^ and hNp55^p.P226L^ caused incomplete regulation on Ca^2+^ transients. Indeed, whereas peak amplitudes were similarly reduced in GCaMP5G-neurons co-expressing either hNp65^p.W135R^ or hNp55^p.P226L^ compared with their wild-type isoform expressing controls, both mutants displayed longer half-width and slower decay times indicating longer Ca^2+^ transients and slower recovery to baseline (Fig. 5B, C). hNp65^p.P342L^ was not as effective as hNp65^wt^ to reduce peak amplitudes (Fig. 5D). Nevertheless, in contrast to hNp65^p.W135R^ and hNp55^p.P226L^, hNp65^p.P342L^ reduced the restoration of basal Ca^2+^ levels similarly to hNp65^wt^ (Fig. 5D). These results indicate that hNp55 is more affected in its functionality than hNp65 by the P226L variant.

### *Nptn* heterozygosity affects PMCA brain levels and social behavior in mice

**Fig. 6:**
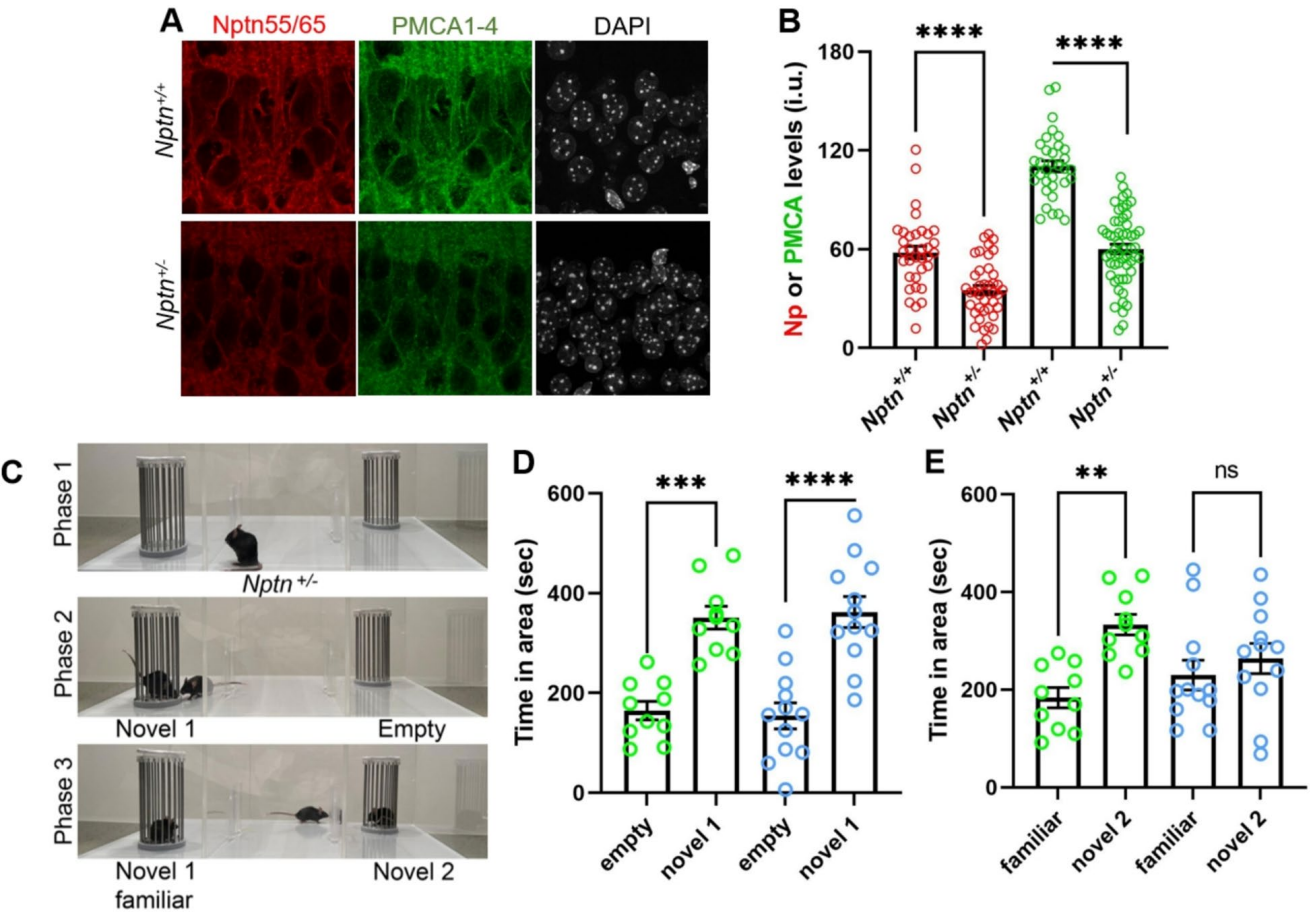
Np-PMCA hypoexpression and social behavior in *Nptn*^+/–^ mice. **A**, **B** Immunoreactivity of anti-Np55/65 (red) and anti-PMCA1-4 (green) antibodies in brain slices from *Nptn*^+*/*+^ and *Nptn*^+/–^ mice was evaluated using confocal microscopy. Nuclei were labelled with DAPI. Maximal projections and fluorescence signal quantification in CA1 hippocampal area showing simultaneous reduction of Np and PMCA expression in *Nptn*^+/–^ mice. **C** The pictures illustrate the experimental phases of the three-chamber test with habituation (Phase 1), exposure to a novel mouse (Phase 2), and exposure to a second novel mouse (Phase 3). *Nptn*^+*/*+^ (*n* = 10, green) and *Nptn*^+/–^ (*n* = 12, blue) mice were measured after habituation (phase 1) in the 3-chamber test. **D** The graph shows the time spent by the tested mouse in the compartments with an unfamiliar mouse (novel 1) or with an empty cup (empty) (Phase 2). The novel mouse is preferred by *Nptn*^+/–^ or *Nptn*^+*/*+^ mice, over an empty cup. **E** *Nptn*^+*/*+^ mice prefer the novel 2 mouse over the familiar mouse, whereas *Nptn*^+/–^ mice displayed no significant preference for novel 2 versus the familiar mouse (Phase 3). Data are presented as mean ± S.E.M. (One-way ANOVA with Scheffe post-hoc; **p < 0.01; ***p < 0.001; ****p < 0.0001; ns: no significant difference)

In mice, the lack of Np results in strongly reduced PMCA levels [6, 11]. Thus, we investigate whether *NPTN* haploinsufficiency as observed in the patients can affect PMCA expression in hippocampal neurons at days 14–16 in vitro (Additional file 1: Fig. S4). A reduction of Np expression by ~ 50% using a pan siRNA against Np55/65 mRNA resulted simultaneously in a partial reduction of PMCA1-4 and increased half-width and decay time of electrically-evoked Ca^2+^ transients, as visualized using GCaMP7s-based Ca^2+^ imaging (Additional file 1: Fig. S4). Furthermore, immunohistochemical evaluation in the hippocampus of 14 days-old *Nptn*^+/–^ mouse pups demonstrated that *Nptn* haploinsufficiency resulted in ~ 50% reduction of Np levels and ~ 45% loss of PMCA1-4 in developing neurons (Fig. 6A,B). Therefore, heterozygous levels of Np^wt^ are insufficient to maintain normal endogenous PMCA levels in the mouse brain.

Autism is a common trait shared by the *NPTN* individuals (Table 1). While general stereotypic behaviors were not observed in heterozygous *Nptn*^+/–^ mice and specifically not detected in the marble burying test (not shown; Additional file 1: Fig. S5), these mice displayed reduced anxiety in the O-maze test (not shown; Additional file 1: Fig. S5). The three-chamber social interaction assay (Fig. 6C) revealed altered social interactions in other mouse models of genetically caused autism [60, 69]. In our experiment, both *Nptn*^+/–^ and *Nptn*^+*/*+^ mice displayed a similar normal preference for a novel mouse compared to an empty cup (Fig. 6D). In contrast to *Nptn*^+*/*+^, which showed the typical higher interest in the stranger over the familiarized mouse, *Nptn*^+/–^ mice did not prefer the second novel over the already familiarized mouse (Fig. 6D). Such reluctance to new social interactions as observed here in *Nptn*^+/–^ mice is indicative for autism-related behavior.

## Discussion

We describe eight individuals with an overlapping, albeit nonspecific, phenotype. All individuals displayed de novo variants in *NPTN* and were diagnosed with autism and/or neurodevelopmental disorders, pointing to the existence of pathological mechanisms related to alterations in *NPTN* that affect neurodevelopment. The identified variants lead to a clinically nonspecific neurodevelopmental disorder with varying severity, although based on a relatively small cohort. We observed that the severity of ID/DD and other clinical findings correlate with the impact on all hNp isoforms (severe) or only on the neuron-specific isoform hNp65 (mild/moderate). This could be tested in mouse mutants that lack only Np65 [65] or all isoforms [11] displaying differences in the severity of their phenotypes (see below). During the writing of this report, additional individuals with *NPTN* variants with neurodevelopmental disorders and autism have been identified. We have, therefore, adopted *NPTN* at the Human Disease Genes series (www.humandiseasegenes.nl) to promote future clinical research on the potential genotype–phenotype correlations.

The nonsense variant of patients 3 and 8 results in loss-of-function with haploinsufficient hNp expression. The frameshift and nonsense variants of patients 4–7 may cause also loss-of-function, as they eventually express truncated proteins produced either without functional domains or in low copy number or without obvious harmful secondary structures. The missense variants of patients 1 and 2 disrupted critical functions of hNp on the regulation of hPMCA levels and cytosolic Ca^2^⁺ dynamics. In particular, the missense variants resulted in mutant hNp expressed in lower amount and with reduced functionality. Both, loss-of-function and missense variants, finally result in insufficient PMCA levels and Ca^2^⁺ signal regulation. Supporting this conclusion, it has been confirmed that Np binds and stabilizes PMCA in protein complexes and promotes the function of PMCA for Ca^2+^ extrusion [6, 11–15, 33, 34]. Furthermore, we show that a decrease in Np levels, due to Np gene deletion or mutation or Np mRNA-interference, results in PMCA reduction in neurons. Importantly, we also show that monoallelic *Nptn* condition results in haploinsufficient production of Np and is insufficient to support normal PMCA levels and led to autism-related social behavior in transgenic mice. Therefore, this report not only confirms prior assumptions regarding the relevance of Np in human neurodevelopment derived from machine learning approaches [70] and correlative genetic studies [10], but also provides a wider perspective to prospective pathological mechanism relevant to other affected individuals, such as those with de novo mutations in PMCA1–4-encoding genes (ATP2B1–4) [21–32].

In our assays, missense variants of the *NPTN* individuals 1 and 2 resulted in decreased production of hNp levels carrying different dysfunctional residues that replaced key conserved amino acids. While the variant of individual 1 substitutes a critical tryptophan within the amino acid sequence at the extracellular and isoform-specific Ig-I of hNp65, individual 2’s variant replaces a proline located at the common transmembrane segment of hNp55 and hNp65 which is embedded into the plasma membrane. The identified binding instability and conformational alterations in these human missense variants indicate that they are either not effectively produced or avidly degraded, as is the case for other Np variants affecting hearing in mice [71, 72]. Our results in HEK293T cells and *Drosophila melanogaster* further support that the *NPTN* variant of patient 2, yielding hNp55^p.P226^ and hNp65^p.P342L^, results in impaired biosynthesis/degradation and is insufficient to support normal PMCA function. This impairment seems to be most detrimental during early development of the flies and is compatible with a dominant negative effect on PMCA function. Interestingly, although human variants from individuals 1 and 2 were expressed at lower levels compared to wt Np isoforms, the *NPTN* Ig-I variant of patient 1 hNp65^p.W135R^ was able to promote PMCA, whereas the *NPTN* transmembrane variant of patient 2 was inefficient in this property. The nonsense variants in individuals 3 and 8 cannot produce hNp proteins and the frameshift leading to premature stop-gain in individuals 4–7 may produce truncated hNp proteins that cannot be anchored to the neuronal plasma membrane and, thus, their effects must result from a diminished capacity to maintain normal PMCA levels. Indeed, we demonstrated that either titrated Np RNAi-targeted knockdown in isolated cultured neurons or monoallelic condition in *Nptn*^+/–^ mice yielded approximately 50% expression levels of Np with a significant reduction in PMCA levels. Ca^2+^ imaging confirmed that a progressive decrease on Np-PMCA content is mirrored by a progressive increase in the Ca^+2^ signal parameters peak amplitude, half width, and decay time. Therefore, we conclude that hNp missense, nonsense, and frameshift variants may result in hypofunctional hNp levels with diminished hNp-PMCA function in neurons.

Insufficient PMCA function may not be the only mechanism underlying the phenotypes of the *NPTN* individuals in this cohort. The missense variant in individual 1 is located at an important extracellular module of hNp65 that mediates homophilic Np65-Np65 *trans*-interactions [56]. In fact, competition of this motif with high-affinity peptides or antibodies destabilizes glutamatergic synaptic contacts and impairs synaptic transmission [73]. As our thermodynamic calculations indicate that this Np65-specific variant displays weakened binding, it is possible to hypothesize the occurrence of an insufficient structural stabilization of excitatory synapses during synaptogenesis in individual 1. Additionally, as reduced synapse formation triggered by Np55 and Np65 occurs in *Nptn*^−/−^ neurons due to failed TRAF6 signaling [59, 73], it is plausible that production of hNp variants in individuals 1 and 2, or haploid wild-type hNp production in individuals 3-to-8, would not trigger Np-TRAF6-dependent synapse formation sufficiently. On the other hand, insufficient hNp production could also alter the excitatory-inhibitory balance affecting development and maturation of neuronal circuits leading to epilepsy or ataxia in the *NPTN* individuals 1-to-3. This idea is based on the requirement of Np for the correct synaptic localization and function of AMPA receptors and α1/2 subunit-containing gamma-aminobutyric acid (GABA) type A receptors [73–77]. Interestingly, recent studies suggest that reduced synaptic transmission involving binding of Np to GABA type A receptors sensitizes rodents to pentylenetetrazole-induced epilepsy [75, 77]. Therefore, hypofunctions and/or malfunctions of other binding partners of hNp such as α-amino-3-hydroxy-5-methyl-4-isoxazolepropionic acid (AMPA) and/or GABA type A receptors may play contributing role in the phenotype of the *NPTN* individuals. These possibilities remain to be evaluated in the future.

Our results in the *Nptn*^+/–^ mice shown here, in combination with our previous reports demonstrating behavioral abnormalities and cognitive deficiencies in constitutive *Nptn*^*−/−*^ mice and inducible Np-deficient mice [11], provide strong evidence for the necessity of Np during brain development. Indeed, induced *Nptn* elimination after normal development demonstrates that Np is acutely required for associative learning and memory in adult mutant mice, while it is not required for other behaviors tested in open field, O-maze, light/dark avoidance learning, light/dark avoidance memory, and startle responses [11]. In contrast, constitutive *Nptn*^*−/−*^ mice fail to perform in all these behavioral tests [11]. Here, we show that haploinsufficient *Nptn*^+/–^ mice with incomplete Np levels display altered social interaction behavior. This confirms our previous results showing impaired social interaction in *Nptn*^*−/−*^, but not in inducible Np-deficient mice, and highlights a strict requirement for normal Np function for successful cognitive development. Furthermore, the archetypical autism-model mouse behavior of reduced preference for a novel *vs.* a familiar mouse in the social interaction test displayed by *Nptn*^+/–^ mice strongly supports the causality of the *NPTN* mutations for the autism diagnosed in all *NPTN* individuals.

The facts that all PMCA are obligatory binding partners of Np and that de novo variants in *ATP2B1* and *ATP2B2* are identified as causing neurodevelopmental disorders including DD/ID and autism [21–27] further strengthens the association of *NPTN*-*ATP2B1-2* and neurodevelopmental disorders. Neuroplastin peripheral malfunctions identified in homozygous Np-deficient mutant mice are not evident in the *NPTN* cohort yet, and, thus they need to be confirmed. Both *Nptn*- and *Atp2b2*-deficient mice and *ATP2B2* individuals are deaf, whereas the *NPTN* cohort described here do not show hearing deficits. This may be attributed to the young age at diagnosis where degeneration of hair cells due to loss of Np-PMCA may not have progressed yet dramatically. Supporting this idea, outer and inner hair cells in the *Nptn*^*−/−*^ cochlea initially develop normally before undergoing degeneration that leads to hearing loss [71]. Alternatively, the residual expression level of Np in our individuals may be sufficient to support hearing and eventually may delay hearing loss to later ages. While neurons express very high levels of Np55 and Np65, most peripheral cell types synthesize only Np55. Altered inflammatory responses by immune cells [12] or impaired pancreatic beta cells function [78] in mice associated to Np55-PMCA deficiency have not been tested nor clinically manifested in the *NPTN* cohort.

Our clinical, animal model, cellular, and molecular data indicate that the de novo variants in *NPTN* are pathogenic and that they affect PMCA expression. We have provided the first evidences in mice connecting *Nptn* expression with normal cognitive capabilities and PMCA expression [6, 11]. Consistently Np-PMCA molecular interaction and its functions in intracellular Ca^2+^ regulation have been extensively confirmed and further detailed [12–15, 33, 34]. Although Desriveres et al*.* using large-scale gene association identified *NPTN* playing a potential role for intellectual deficits in humans [10], and Dhindsa et al*.* using a machine learning approach based on gene constraint, expression, and other gene-level annotations, predicted *NPTN* as preferably causing an autosomal dominant neurodevelopmental condition (percentiles of 90.8 for DD, 94.0 for ASD, and 97.9 for developmental and epileptic encephalopathy) [70], more direct evidence for a critical relevance of *NPTN* for brain development was missing. To our knowledge, this is the first report linking *NPTN* to human neurodevelopment.

## Conclusions

In summary, we establish that de novo variants in *NPTN* as causative for a neurodevelopmental disorder and autism. Based on several lines of evidence shown here and also in the literature, we proposed that impaired or insufficient function of the Np-PMCA complexes may contribute to the *NPTN* disorder.

## Supplementary Information

Additional file 1.

Additional file 2.

## Data Availability

Identified variants in NPTN have been uploaded to ClinVar https://www.ncbi.nlm.nih.gov/clinvar/submitters/506086/. Other datasets used and/or analyzed during the current study are available from the authors on reasonable request.
