# Supplementary material for: De novo variants in NPTN cause a neurodevelopmental disorder with autism and neuroplastin-PMCA hypofunction
Source: Genome Med. 2026 Jul 1;18:93. doi: 10.1186/s13073-026-01699-7 (PMC13321906; doi:10.1186/s13073-026-01699-7)
Supplement: Supplementary file 1 — Additional file 1. [file 13073_2026_1699_MOESM1_ESM.docx]

[**Case reports 2**](#_Toc188971396)

[**Individual 1, c.403T>A (p.Trp135Arg), *de novo* 2**](#_Toc188971397)

[**Individual 2, c.1025C>T (p.Pro342Leu), *de novo* 3**](#_Toc188971398)

[**Individual 3, c.14C>A (p.Ser5*), *de novo* 4**](#_Toc188971399)

[**Individual 4, c.218_219del (p.Glu73Valfs*53), *de novo*, mosaic 5**](#_Toc188971400)

[**Individual 5, c.284C>G (p.Ser95*), heterozygous 6**](#_Toc188971401)

[**Individual 6, c.342C>G (p.Tyr114*), *de novo* 7**](#_Toc188971402)

[**Individual 7, c.902del (p.Asn301Thrfs*3), *de novo***](#_Toc188971403) **8**

## **Individual 8, c.14C>A (p.Ser5*), *de novo*..………………………………………………………………………….9**

[**Supplementary methods 10**](#_Toc188971404)

[**Supplementary figures 12**](#_Toc188971408)**2**

[**Supplementary**](#_Toc188971409) [**tables 1**](#_Toc188971417)**7**

[**Supplementary**](#_Toc188971409) [**references**](#_Toc188971417) **20**

# **Case reports**

## **Individual 1, c.403T>A (p.Trp135Arg), *de novo***

Patient 1 is a female with moderate intellectual disability, autism spectrum disorder, and epilepsy. She was the first child born to non-consanguineous parents with an unremarkable family history. The pregnancy was uncomplicated, and she was delivered at term by Caesarian section due to failure to progress. Parents first became concerned at age 4 months due to slow developmental progress. She developed epileptic spasms and focal impaired awareness seizures at 7 months. She was placed on steroids for two months. Additional antiseizure medications included vigabatrin, phenobarbital, levetiracetam, and topiramate. She was maintained on topiramate until 4 years old and then weaned off. She remained seizure-free until 8 years old, when she began having partial focal seizures. She is treated with oxycarbazepine but still has occasional breakthrough seizures. She had global developmental delays, with independent walking achieved by 2.5 years and first words between 3-4 years. She was diagnosed with autism spectrum disorder at age 9 years. Neuropsychological testing at age 11 years revealed a full-scale IQ of 40 and adaptive functioning consistent with moderate ID. She has myopia and intermittent exotropia. At her most recent clinical evaluation at 17 years, her height was 158 cm (22 %ile, -0.77 SD), weight was 75.8 kg (93 %ile, +1.48 SD), and head circumference was 56 cm (81 %ile, +0.88 SD). She has subtle dysmorphic facial features, including upslanting palpebral fissures, slight ectropion, a prominent forehead, frontal upsweep, and a high anterior hairline. Neurologic exam was normal. She communicates verbally and receives special education support in an inclusive school setting. Several brain MRIs have been unremarkable.

## **Individual 2, c.1025C>T (p.Pro342Leu), *de novo***

The proband is the second child of non-consanguineous parents of German and Polish descent. After an unremarkable prenatal history, the proband was born by Caesarean section at 40+2 weeks gestational age with unremarkable birth measurements. Early childhood development was unremarkable. Free walking was possible at 12 months, and he also spoke his first words at 12 months. From the age of 30 months, the proband stopped speaking completely and has only imitated sounds since then. He was noted to have limited social interactions and was diagnosed with autism spectrum disorder. At age 6 years old, the proband began to develop abnormal eye movements with rapid eye fluttering every second to third day. A brain MRI and EEG were completed and reported as normal. A suspected diagnosis of complex focal seizures was made (DD: tic disorder, dystonia). Anticonvulsant drug therapy was not initiated. He was approved for speech therapy, occupational therapy, and autism-specific therapy. On the physical exam, the proband showed subtle dysmorphisms with mild hypertelorism, downslanting palpebral fissures, full lips, Darwin tuberculum on both ears, fetal pads, and sandal gaps.

## **Individual 3, c.14C>A (p.Ser5*), *de novo***

Patient 3 is the first child of a French non-consanguineous couple. The pregnancy was unremarkable apart from non-insulin-dependent gestational diabetes. She was born at 42 weeks of postmenstrual age with normal birth parameters (weight 3170g; length 49 cm; head circumference 33.5 cm). The neonatal period was uneventful. She presented with psychomotor delay noted by the parents around 8 months old. She was able to sit unsupported at 14 months and sit alone at 19 months, crawl on all fours at 16 months, and walk unaided at 26 months. She started babbling around 1 year old and saying “mama” but in an undirected fashion. Expressive language didn’t progress, and she still uses syllables at 5 years old. She is able to understand simple orders and to use pictograms to communicate. She learned to sign a few words. Progress remains very slow; her evolution is that of severe intellectual disability. Autistic traits were noted as early as 18 months, including poor eye contact, stereotypic movements, and, later, an eating disorder. Autism spectrum disorder was confirmed after specific evaluation. She had 2 brain MRI identifying cerebellar dysgenesis located in the superior part, with entwined cerebellar furrows. She presented with recurrent pyelonephritis secondary to vesicoureteral reflux.

## **Individual 4, c.218_219del (p.Glu73Valfs*53), *de novo*, mosaic**

The proband is a 6-year-old male. He is the third child of nonconsanguineous parents. He presents with autism spectrum disorder (ASD), developmental regression, speech and language delay, developmental delay in social communication skills, and repetitive/stereotyped behaviors. He was born at 40 weeks gestation after an unremarkable pregnancy and went home after 2-3 days. He began to speak some words by 1 year. After he began to speak, he experienced a speech regression at approximately 2 years old, losing several words that he had learned between 18 and 24 months. The episode lasted about 4 months. Between 18 months and 2 years, he began to exhibit repetitive and stereotyped behaviors during play. He was enrolled in early intervention (EI) and was subsequently referred for an autism evaluation. He was diagnosed with ASD around the age of 2 years and 10 months. He receives ABA, occupational, and speech therapies and has an IEP for behavioral support. Currently he is using an AAC device to communicate and has about 10 words and several signs. He continues to make slow progress in speech and enhancing social skills. Additional medical history includes allergies, eczema, and hyperopic astigmatism in both eyes.

## **Individual 5, c.284C>G (p.Ser95*), heterozygous**

The proband was born premature at 29 w 6d. Pregnancy and postnatal period were additionally complicated by *in utero* polysubstance exposure and a two-month NICU stay. She reportedly crawled at 7 or 8 months and walked at 18 months. At age 3 years, she says a few single words and has several signs. She was diagnosed with autism spectrum disorder. The proband received early intervention therapies, including physical, occupational, and speech therapy. She is reportedly meeting expectations socially and academically in preschool and will be starting kindergarten in a typical classroom without plans for special education provisions. She has a history of torticollis. She required surgery twice for laryngomalacia. At age 20 months, the proband presented to neurology due to concerns about starting episodes that reportedly had been present since birth. An EEG was attempted but was unsuccessful due to poor patient cooperation. Brain MRI was normal. Anti-seizure medications were initiated by the proband’s neurologist. She additionally presented with tone abnormalities as well as some hyperreflexia. On physical examination, she has no dysmorphic features.

## **Individual 6, c.342C>G (p.Tyr114*), *de novo***

The proband had an unremarkable prenatal and birth history, being born at 38 weeks gestational age, vaginally, as the product of a non-consanguineous union between parents of Puerto Rican and Puerto Rican/Irish descent. He was developing appropriately and did not have medical issues beyond eczema and early eruption of his teeth starting at 8 months old. By 10 months old, the proband was noted to have limited social interactions. He began to have sensory behavioral issues (hand flapping, head banging), hyperactivity, and toe walking. The proband was evaluated by Early Intervention at 18 months old and was diagnosed with a speech delay, qualifying for speech therapy and special instruction. His hearing evaluation was within normal limits. By 21 months old, he was diagnosed with autism spectrum disorder and was approved for physical therapy, occupational therapy, and ABA services (applied behavior analysis). At 20 months old, the proband began to develop abnormal eye movements with rapid eye fluttering. He also developed insomnia. He was evaluated by Neurology when he was 2 years old. Recommendations included an EEG and brain MRI, which the proband could not tolerate. At 26 months old, a 24-hour EEG was performed, which was normal. A brain MRI was completed and was normal as well. Since starting services, the proband has had some improvements in his behavior, though he remained primarily non-verbal and hyperactive.

On the physical exam, the proband was mildly dysmorphic with a prominent forehead and deep-set eyes. He did have areas of hyperpigmentation over his body, mostly on the left side of his body.

## **Individual 7, c.902del (p.Asn301Thrfs*3), *de novo***

This boy was born after an uneventful pregnancy at 39;2 weeks of gestation, with a birth weight of 3725 grams (70^th^ percentile). His Apgar score was 9/10. His neonatal period was uneventful. His development was significantly delayed: he walked unaided at 19 months of age and spoke his first words at 3 years of age. At the age of 9 years, he only used 4 single words. He had normal hearing and eyesight and a normal day-night rhythm. At the age of 5 years, auto-mutilation started, and it also seemed that he had a high pain threshold. At the age of 8 years, his level of functioning was officially assessed at the level of 13 months of age, with an emotional development under 6 months of age. He developed pica behavior. At 9 years of age, he was seen in the clinical genetics department. He had a height of 132 cm (15^th^ centile), a weight of 30 kg (75^th^ centile) and a head circumference of 52.8 cm (50^th^ centile). He had a broad flat face with full eyebrows, epicanthal folds, upward slanting palpebral fissures, large everted ears, broad mouth with a full lower lip, broad dental gums. He had relatively long fingers with minor clinodactyly of the 5^th^ fingers and feet with sandal gaps. Both parents were unrelated and normally educated and originated from China.

## **Individual 8, c.14C>A (p.Ser5*), *de novo***

The proband is the first and only child of non-consanguineous parents of Algerian origin. After an uneventful prenatal history, she was born at 38 weeks’ gestation by cesarean section due to fetal macrosomia. Her birth weight was 4.25 kg, length 51 cm, and head circumference 36 cm. Early childhood development was normal: she sat unsupported at 9 months and walked independently at 12 months.

Concerns arose at age 3 due to slow developmental progress, as well as learning and comprehension difficulties. She exhibited overgrowth; at 7 years old, her weight was 65.6 kg, height 125 cm, and head circumference 55.5 cm (+3 SD). Neurological examination was normal. She communicates verbally and has subtle dysmorphic facial features, including hypertelorism and epicanthus. Her fingers are short. She suffers from sleep apnea. She currently attends a mainstream school with educational support and receives speech therapy.

# **Supplementary Methods**

## **Table A:** Primers

| FUGW-hUbC-Fw | 5’- CTGAAGCTCCGGTTTTGAAC -3’ |
| --- | --- |
| FUGW-vorLTR-Rev | 5’- CGTTGGGAGTGAATTAGCC -3’ |
| hNp65 WT-Fw | 5’- GACTCTAGAGGATCCAGGATGTCGGGTTCGTCGCTGCC -3’ |
| hNp65 WT-Rev | 5’- GTTTTTCTAGGTCTCGAGTTAATTTGTGTTTCTCTGGCGC- 3’ |
| hNP65 P342L-Fw | 5’- GCCCCACTCTGGCTTTTCTTGGGAATT -3’ |
| hNP65 P342L-Rev | 5’- AATTCCCAAGAAAAGCCAGAGTGGGGC -3’ |
| hNP65 W135R-Fw | 5’- CCCTCCATAACAAGGATTCGAGCCCAGGCCACC -3’ |
| hNP65 W135R-Rev | 5’- GGTGGCCTGGGCTCGAATCCTTGTTATGGAGGG -3’ |

## **Table B:** Antibodies

| Antibodies | Host | Dilution | Company (Cat. #) |
| --- | --- | --- | --- |
| Primary | | | |
| α-Neuroplastin | Sheep | IHC: 1:300/500 WB: 1:5,000 | RD systems (AF5174) |
| α-β-Actin | Mouse | WB: 1:10,000 | Thermo Fisher (A5441) |
| α-GFP | Mouse | WB: 1:10,000 | Sigma (11814460001) |
| α-PMCA | Rabbit | IHC: 1:300 | Thermo Fisher (MA3-914) |
| Secondary | | | |
| α-sheep Cy3 | Donkey | IHC: 1:300/1000 | Jackson ImmunoResearch Lab. (713-165-147) |
| α-rabbit Alexa 488 | Donkey | IHC: 1:300 | Jackson ImmunoResearch Lab. (715-175-152) |
| α-Hrp/ Alexa 647 | Goat | IHC: 1:300 | Jackson ImmunoResearch Lab. (123-605-021) |

## **Generation and imaging of mutant *Drosophila melanogaster***

Transgenic flies carrying both a Gal4-inducible UAS-bsg-RNAi construct (on third chromosome) and either the Gal4-inducible UAS-hNp55 or UAS-hNp55^p.P342L^ construct, recombinant strains were established, taking advantage of traceable dominant markers associated with balancer chromosomes. The recombined *Drosophila* lines generated according to the following work flow as follows:

Only the second and third chromosomal genotypes are depicted, separated by semicolon. Marker and balancer chromosomes include Bl^1^ (recognizable as shortened bristles), CyO^GFP^ (recognizable by green fluorescence and by curled wings), TM2 (recognizable by swollen halteres) and TM6 (recognizable by tubby appearance during larval/pupal stages and by super nummary bristles in the humeral part of the adult thorax). Segregations of these markers was used as a criterion to select flies with the desired genotypes. The UAS-hNp55^(WT or p.P342L)^ /CyO^GFP^; UAS-bsg-RNAi/TM6 recombinant flies (red box) were established as a stable stock, from which males or females were crossed to the muscle-specific Gal4-activator line *C57-Gal4* (blue box). *Drosophila* C57-Gal4 stocks are as follow: Control w1118 (control in main text), Bsg RNAi^GD2789^ (Bsg KD), Bsg RNAi^GD2789^+ hNp55^WT^ rescue (Np55^WT^), Bsg RNAi^GD2789^+ hNp55^p.P>L^ rescue (hNp55^p.P342L^).

# **Supplementary Figures**

**
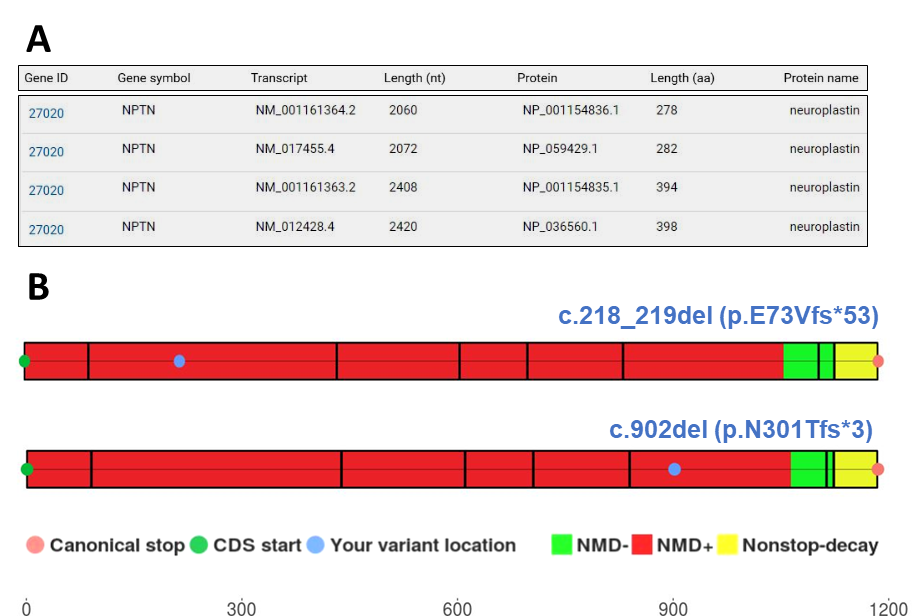
**

**Fig. S1.** *NPTN* frameshift variants and nonsense-mediated mRNA decay. (**A**) Access numbers of mRNA transcripts and protein products encoded by *NPTN* were extracted from <https://www.ncbi.nlm.nih.gov/datasets/gene/id/27020/products/> (**B**) The *NPTN* frameshift variants c.218_219del (p.E73Vfs*53) and c.902del (p.N301Tfs*3) producing a nonsense stop-codon were submitted to nonsense-mediated mRNA decay analysis using the publicly available nmdescpredictor tool**^1-3^** and identified as “subject to degradation by nonsense-mediated decay”**^1-3^** <https://nmdprediction.shinyapps.io/nmdescpredictor/.>

**
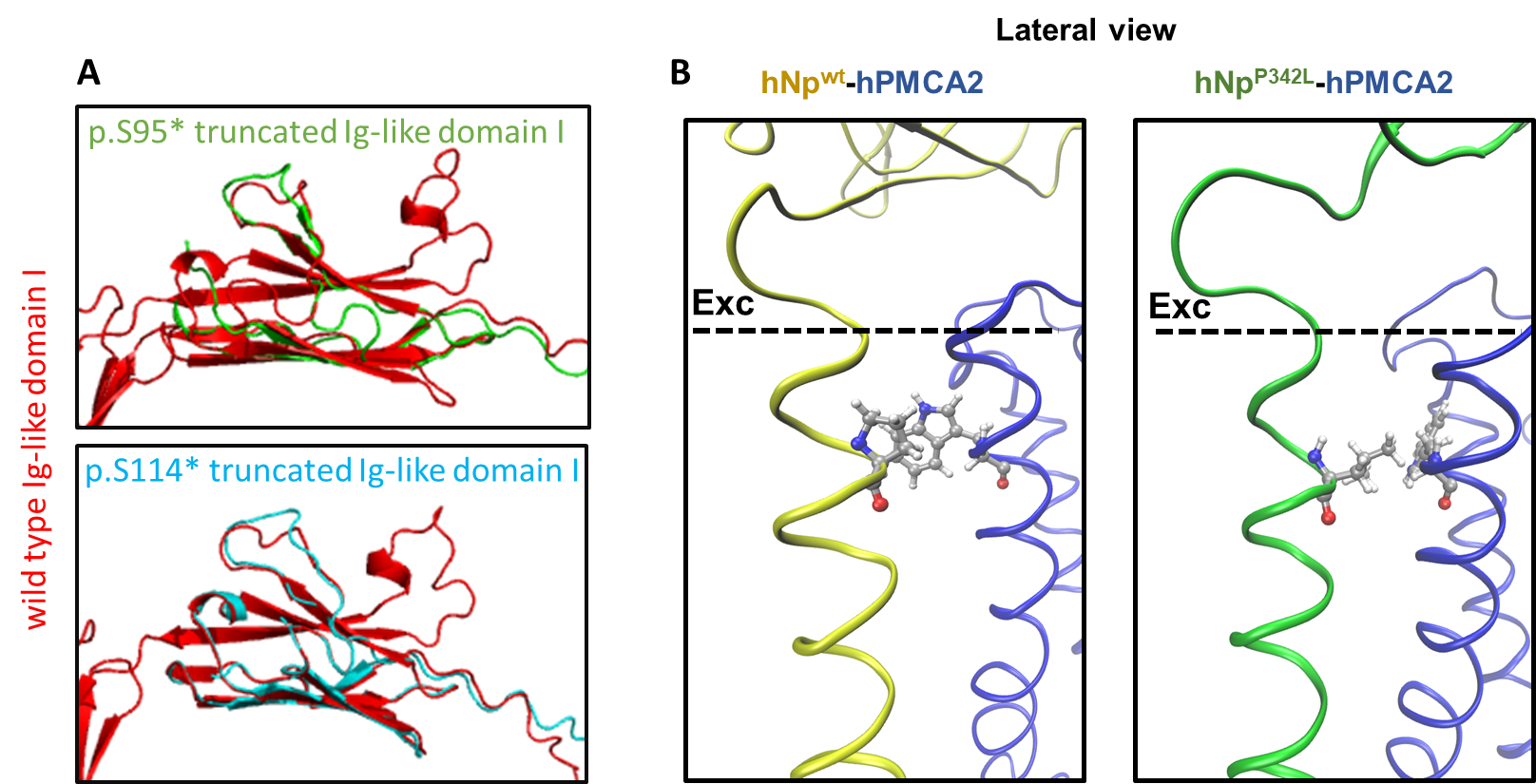
**

**Fig. S2.** *In silico* analysis of truncated and mutant hNp protein variants. (**A**) hNpp.S95* and hNpp. T114* may be produced as truncated Ig-like I domains with antiparallel *β*-sheet structures and without the capacity to form a folded Ig-like structure. (**B**) Confirmation of the conserved interface for the hNpp.P342 (hNp^wt^ in yellow, left panel) and hPMCA2p.W1043 (blue, left panel) was reproduced using high-resolution PMCA2-NPTN cryo-EM structure (Vinayagam *et al*.**^7^**). This interaction is perturbed in the mutant interface hNpp.P342L- hPMCA2p.W1043 (right panel). Exc, extracellular.

**
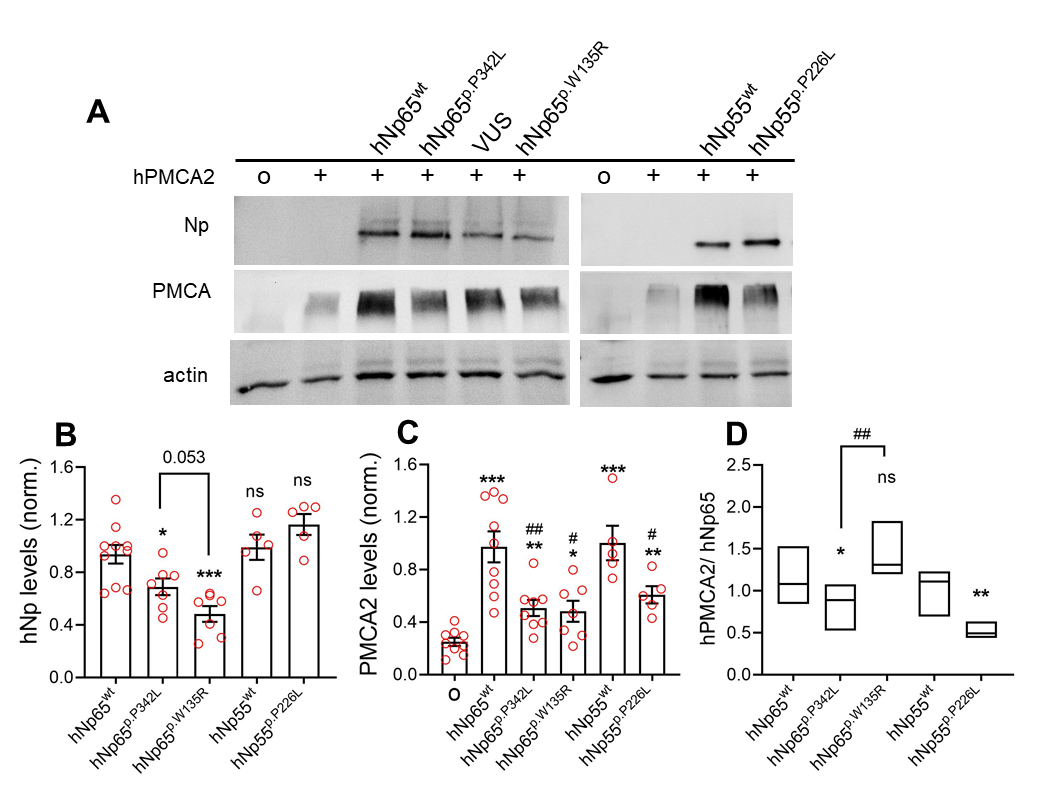
**

**Fig S3.** Expression of *NPTN* missense variants in HEK cells. (A) Western blot analysis of protein homogenates from HEK293T cells transiently co-transfected with hPMCA2 and either hNp65^wt^, hNp55^wt^ or one of the missense variants hNp65^p.P342L^, hNp65^p.W135R^ or hNp55^p.P226L^. Negative control for co-transfections and antibody specificity (o) and control for single hPMCA2 expression are included. (B, C) Quantifications of Western blot analysis. Densitometric values Np and PMCA2 bands were corrected with actin values and normalized to hNp55^wt^. For Np levels: hNp65^wt^ (n = 10; mean = 0.94:SD=0.22), hNp65^p.P342L^ (7; 0.69:0.17), hNp65^p.W135R^ (7; 0.48:0.16), hNp55^wt^ (5; 1.00:0.21), hNp55^p.P226L^ (5; 1.17:0.18). Unless indicated otherwise, *p < 0.05, ***p < 0.001 or no significant difference (ns) *vs* the corresponding wt isoform using Mann-Whitney test. For PMCA2 levels hNp65^wt^ (mean = 0.97:SD=0.35), hNp65^p.P342L^ (0.51:0.17), hNp65^p.W135R^ (0.48:0.21), hNp55^wt^ (1.00:0.34), hNp55^p.P226L^ (0.61:0.14). * p < 0.05, **p < 0.01, ***p < 0.001 vs hPMCA2 (o) using Mann-Whitney test. #p < 0.05 or ##p < 0.01 *vs* the corresponding wt isoform. (D) hPMCA2/hNp65 ratio was calculated using values of Np in B and hPMCA2 in C and displayed as box diagram where the median is shown. Unless indicated otherwise, *p < 0.05 or **p < 0.01 *vs* the corresponding wt isoforms using Mann-Whitney test. VUS = variant of unknown significance.

**
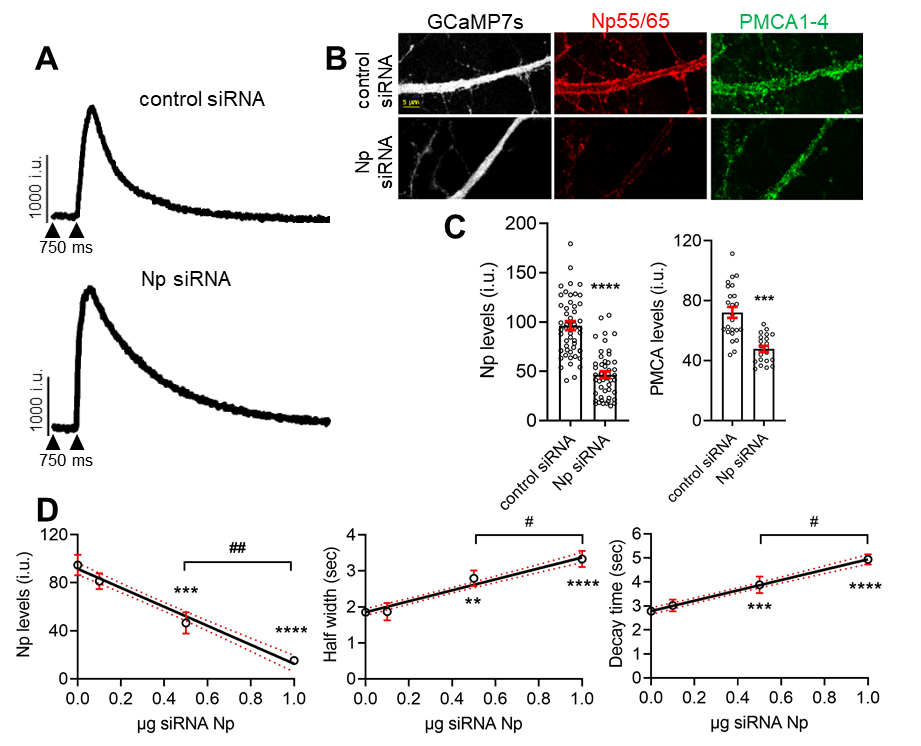
**

**Fig S4.** Decreased levels of Np^wt^ results in PMCA loss and correlates with inefficient cytosolic Ca^2+^ regulation. (**A**) Examples of raw traces of electrically-evoked Ca^2+^ transients in GCaMP7S-expressing mouse neurons, co-transfected with either control (scramble) siRNA- or Np siRNA and stimulated with 20 pulses at 20 Hz. (**B**) Confocal images of GCaMP7S-expressing dendritic segments of neurons co-transfected with control (scramble) siRNA- or Np siRNA (0.4 µg) were taken after Ca^2+^ imaging, fixation, and staining using pan anti-Np55/65 (red) and pan anti-PMCA1-4 antibodies (green). (**C**) Quantification of endogenous Np- and PMCA-associated fluorescent signal (intensity units, i.u.) in the somatodendritic compartment of GCaMP7S-expressing neurons co-transfected or not with control (scramble) siRNA- or Np siRNA (0.4 µg) is shown for simplicity as mean±S.E.M. from four experimental groups but mean±S.D. are also given later for each condition. For Np levels ****p < 0.0001 unpaired *t*-test between control siRNA (n = 54; mean: 96.40 ± S.D.:32.95) and Np siRNA (48; 46.57 ± 23.82). For PMCA levels ***p < 0.001 unpaired t-test between control siRNA (n = 28; mean: 72.19 ± S.D.:17.82) and Np siRNA (24; 47.82 ± 9.383). (**D**) Correlative analysis between total Np levels, half-width, or decay time of the evoked Ca^2+^ transients in dendrites *vs* µg of siRNA Np. Open circles are mean±S.E. from at least three independent experiments. For Np levels ***p < 0.001 or ****p < 0.0001 unpaired t-test between 0.0 µg (n = 50; mean: 94.72 ± S.D.:29.94) and 0.5 µg (30; 45.57 ± 23.82) or 1.0 µg (35; 15.21 ± 4.7). For half-width **p < 0.01 or ****p < 0.0001 unpaired *t*-test between 0.0 µg (n = 436; mean: 1.85 ± S.D.:0.62) and 0.5 µg (122; 2.79 ± 1.21) or 1.0 µg (35; 3.33 ± 1.73). For decay time ***p < 0.001 or ****p < 0.0001 unpaired t-test between 0.0 µg (mean: 2.78 ± S.D.:1.62) and 0.5 µg (3.88 ± 1.92) or 1.0 µg (4.94 ± 1.65).


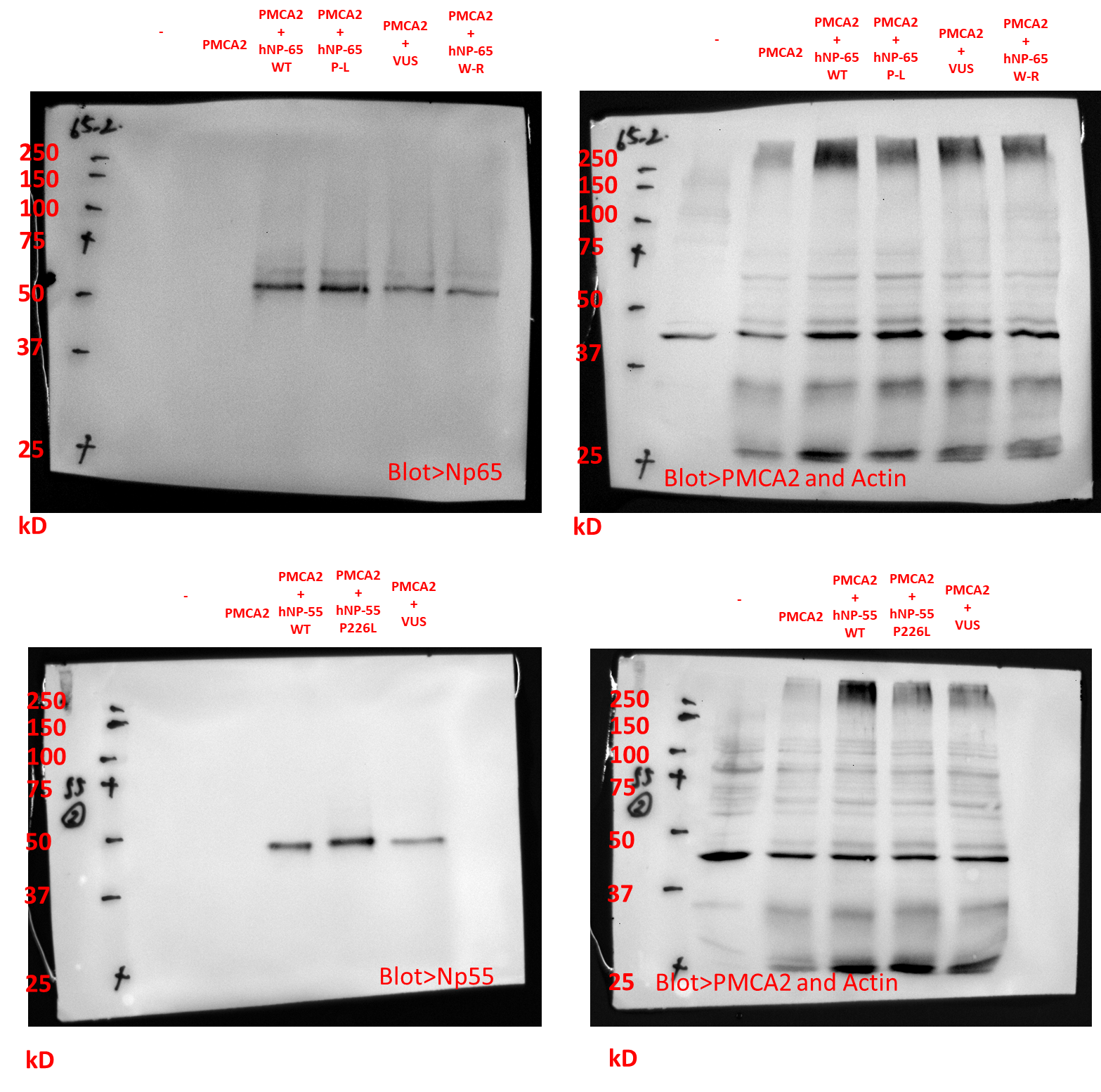


**Supplementary tables**

**Table S1.** Variant information and classification according to ACMG criteria**^3^**. NMD, nonsense mediated mRNA decay.

| **individual** | **Chr15: g.(hg38)** | **c.** | **p.** | **allelic state** | **origin** | **predicted effect /**  **Np isoform affected** | **ACMG criteria** | **classification** |
| --- | --- | --- | --- | --- | --- | --- | --- | --- |
| 1 | 73597058 | 403T>A | Trp135Arg | heterozygous | de novo | missense  Np65 | PS2_MOD, PS3, PM2_SUP, PP3 | likely pathogenic |
| 2 | 73570239 | 1025C>T | Pro342Leu | heterozygous | de novo | missense  Np65 and Np55 | PS2_MOD, PS3, PM2_SUP, PP3 | likely pathogenic |
| 3 | 73633202 | 14C>A | Ser5* | heterozygous | de novo | No mRNA translation  Np65 and Np55 | PVS1, PS2_MOD, PM2_SUP | pathogenic |
| 4 | 73597244_73597245del | 218_219del | Glu73Valfs*53 | heterozygous  mosaic | de novo | NMD  Np65 | PVS1, PS2_MOD, PM2_SUP | pathogenic |
| 5 | 73597177 | 284C>G | Ser95* | heterozygous | unknown | NMD  Np65 | PVS1, PM2_SUP | likely pathogenic |
| 6 | 73597119 | 342C>G | Tyr114* | heterozygous | de novo | NMD  Np65 | PVS1, PS2_MOD, PM2_SUP | pathogenic |
| 7 | 73570363del | 902del | Asn301Thrfs*3 | heterozygous | de novo | NMD  Np65 and Np55 | PVS1, PS2_MOD, PM2_SUP | pathogenic |
| 8 | 73633202 | 14C>A | Ser5* | heterozygous  mosaic | de novo | No mRNA translation  Np65 and Np55 | PVS1, PS2_MOD, PM2_SUP | pathogenic |

**Table S2.** *In silico* prediction of missense variants in *NPTN*. *In silico* scores were retrieved using dbNSFP**^4^**. Cutoffs for *in silico* scores were derived from Pejaver *et al.***^5^** Red color signifies damaging prediction (at least PP3_SUP score in Pejaver *et al.***^5^** was reached); yellow color signifies neither PP3_SUP nor BP4_SUP score was reached; green color signifies benign prediction (BP4_SUP score in Pejaver *et al.* **^5^**). For further annotations on the missense variants, see Supplementary table 4.

| **Individual** | **Chr15: g.(hg38)** | **c.** | **p.** | **CADD-v1.6** | **REVEL** | **MutPred2** | **VEST4** | **BayesDel** | **AA conservation** | **gnomAD v4** |
| --- | --- | --- | --- | --- | --- | --- | --- | --- | --- | --- |
| 1 | 73597058 | 403T>A | Trp135Arg | 30.0 | 0.607 | 0.557 | 0.829 | 0.31 | high, up to *C. elegans* | 0 |
| 2 | 73570239 | 1025C>T | Pro342Leu | 29.7 | 0.594 | 0.686 | 0.805 | 0.25 | high, up to *C. elegans* | 0 |

**Table S3.** Detailed clinical data of individuals with causative variants in *NPTN.* See separate Excel file (Additional file 2: Table S3).

**Table S4.** Annotation and *in silico* scores of all missense variants in *NPTN.* All annotations were retrieved using dbNSFP v4.5.**^4^** (Ref: doi: 10.1186/s13073-020-00803-9). See separate Excel file (Additional file 2: Table S4).

**Table S5.** Behavioural tests in *Nptn^-/-^* or *Nptn^+/-^* mice *vs*. *Nptn^+/+^ mice.* An asterisk (*****) indicates data published**^6^**. Other data are included in this report as indicated. (**#**) Diogenes-like behavior describes self-centered behavior, e.g. upon cage lid opening, in open mazes, etc. mice display a "leave me alone attitude".

| **Test** | ***Nptn^-/-^*** | ***Nptn^+/-^*** |
| --- | --- | --- |
| General inspection | Diogenes-like behavior^(^**^#^**^)^ | Normal |
| Open field | More in center ^(^**^*^**^)^ | No difference ^(^**^*^**^)^ |
| O-maze | No difference ^(^**^*^**^)^ | Reduced anxiety |
| Light-dark (LD) avoidance | More transitions and time in light ^(^**^*^**^)^ | Normal, not shown |
| Social interaction | Less novel/ familiar^(^**^*^**^)^ | Less novel/ familiar, Fig. 6 |
| Startle response | Less startle response ^(^**^*^**^)^ | Less startle response ^(^**^*^**^)^ |
| Pre-pulse inhibition | No PPI^(*)^ | Less PPI^(*)^ |
| Tail suspension | Less mobility ^(*)^ | Not determined |
| Aggression | Normal, not shown | Not determined |
| Marble burying | Not determined | Normal, not shown |

**Supplementary references**

1. Coban-Akdemir Z, White JJ, Song X, Jhangiani SN, Fatih JM, Gambin T, Bayram Y, Chinn IK, Karaca E, Punetha J, Poli C; Baylor-Hopkins Center for Mendelian Genomics; Boerwinkle E, Shaw CA, Orange JS, Gibbs RA, Lappalainen T, Lupski JR, Carvalho CMB. Identifying Genes Whose Mutant Transcripts Cause Dominant Disease Traits by Potential Gain-of-Function Alleles. Am J Hum Genet. 2018 Aug 2;103(2):171-187. doi: 10.1016/j.ajhg.2018.06.009.
2. Lucia Laugwitz, Fubo Cheng, Stephan C Collins, Alexander Hustinx, Nicolas Navarro, Simon Welsch, Helen Cox, Tzung-Chien Hsieh, Aswinkumar Vijayananth, Rebecca Buchert, Benjamin Bender, Stephanie Efthymiou, David Murphy, Faisal Zafar, Nuzhat Rana, Ute Grasshoff, Ruth J Falb, Mona Grimmel, Annette Seibt, Wenxu Zheng, Hamid Ghaedi, Marie Thirion, Sébastien Couette, Reza Azizimalamiri, Saeid Sadeghian, Hamid Galehdari, Mina Zamani, Jawaher Zeighami, Alireza Sedaghat, Samira Molaei Ramshe, Ali Zare, Behnam Alipoor, Dirk Klee, Marc Sturm, Stephan Ossowski, Henry Houlden, Olaf Riess, Dagmar Wieczorek, Ryan Gavin, Reza Maroofian, Peter Krawitz, Binnaz Yalcin, Felix Distelmaier, Tobias B Haack, Genomics England Research Consortium, ZSCAN10 deficiency causes a neurodevelopmental disorder with characteristic oto-facial malformations, Brain, Volume 147, Issue 7, July 2024, Pages 2471–2482, <https://doi.org/10.1093/brain/awae058>
3. Richards S, Aziz N, Bale S, Bick D, Das S, Gastier-Foster J, Grody WW, Hegde M, Lyon E, Spector E, Voelkerding K, Rehm HL; ACMG Laboratory Quality Assurance Committee. Standards and guidelines for the interpretation of sequence variants: a joint consensus recommendation of the American College of Medical Genetics and Genomics and the Association for Molecular Pathology. Genet Med. 2015 May;17(5):405-24. doi: 10.1038/gim.2015.30
4. Liu X, Li C, Mou C, Dong Y, Tu Y. dbNSFP v4: a comprehensive database of transcript-specific functional predictions and annotations for human nonsynonymous and splice-site SNVs. Genome Med. 2020 Dec 2;12(1):103. doi: 10.1186/s13073-020-00803-9. PMID: 33261662; PMCID: PMC7709417.
5. Pejaver V, Byrne AB, Feng BJ, Pagel KA, Mooney SD, Karchin R, O'Donnell-Luria A, Harrison SM, Tavtigian SV, Greenblatt MS, Biesecker LG, Radivojac P, Brenner SE; ClinGen Sequence Variant Interpretation Working Group. Calibration of computational tools for missense variant pathogenicity classification and ClinGen recommendations for PP3/BP4 criteria. Am J Hum Genet. 2022 Dec 1;109(12):2163-2177. doi: 10.1016/j.ajhg.2022.10.013
6. Bhattacharya S, Herrera-Molina R, Sabanov V, Ahmed T, Iscru E, Stöber F, Richter K, Fischer KD, Angenstein F, Goldschmidt J, Beesley PW, Balschun D, Smalla KH, Gundelfinger ED, Montag D. Genetically Induced Retrograde Amnesia of Associative Memories After Neuroplastin Ablation. Biol Psychiatry. 2017 Jan 15;81(2):124-135. doi: 10.1016/j.biopsych.2016.03.2107.
7. Vinayagam D, Sitsel O, Schulte U, Constantin CE, Oosterheert W, Prumbaum D, Zolles G, Fakler B, Raunser S. Molecular mechanism of ultrafast transport by plasma membrane Ca2+-ATPases. Nature. 2025 Oct;646(8083):236-245. doi: 10.1038/s41586-025-09402-3. Epub 2025 Aug 20. PMID: 40836084; PMCID: PMC12488499.
